# Mechanistic Insights into GTP Hydrolysis by the RhoA Protein: Catalytic Impact of Glutamine Tautomerism

**DOI:** 10.1021/acscatal.5c00719

**Published:** 2025-02-28

**Authors:** Jorge Pardos, Adrián García-Martínez, J. Javier Ruiz-Pernía, Iñaki Tuñón

**Affiliations:** Departamento de Química Física, 16781 Universidad de Valencia, 46100 Burjassot, Spain

**Keywords:** GTP hydrolysis, Ras proteins, QM/MM, free energy, amide–imide tautomerism

## Abstract

We present a systematic evaluation of different possible reaction mechanisms for GTP hydrolysis in RhoA, a member of the Ras superfamily of enzymes that uses this reaction to switch from an active to an inactive conformation. These enzymes are activated by the presence of a GTPase activating protein (or GAP) that forms an intimate complex with residues of the two proteins present in the active site. We have explored the multidimensional reactional free energy landscape in the active site of the complex formed by RhoA and p50RhoGAP. Our molecular dynamics simulations show that the activating enzyme p50RhoGAP establishes catalytically important interactions with the phosphate groups of GTP through its so-called arginine finger (Arg85) and also with the RhoA residue Gln63. This is a key residue because it not only interacts with the nucleophilic water molecule but also participates actively in the reaction mechanism. Adaptive string method simulations using hybrid quantum mechanics/molecular mechanics (QM/MM) potentials with both tight-binding and density functional Hamiltonians show that GTP hydrolysis proceeds through the formation of a metaphosphate metastable species. Mechanistic proposals differ in the proton transfer rearrangements required to form the inorganic phosphate ion. Our simulations discard a solvent-assisted mechanism and point to the participation of Gln63 in the proton transfer process by means of the side chain tautomerism from the amide to the imide form. The proton transfer required to recover the amide form of Gln63 requires the participation of the inorganic phosphate, and it is the rate-limiting step of the process, with a free energy barrier of 20.2 kcal mol^–1^ at the B3LYPD3/MM level, in good agreement with the experimentally derived value. The amide–imide tautomerism could also be relevant in other enzymes, facilitating proton transfer events in complex mechanisms.

## Graphical abstract

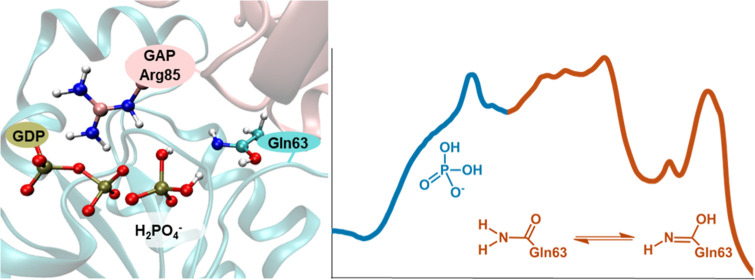

## 1 Introduction

The Ras superfamily of guanine nucleotide-binding proteins is involved in diverse cellular processes, including cell survival, cell cycle progression, cell polarity, cell movement, actin cytoskeletal organization or vesicular and nuclear transport.^1,2^ These proteins act as molecular switches, cycling between a biologically active state, bound to guanosine triphosphate (GTP), and a biologically inactive state, bound to guanosine diphosphate (GDP).^3–5^ Members of the Ras superfamily share a unique structural domain known as the G domain, which consists of a central β-sheet with 6 strands surrounded by 5 α-helices on both sides and is responsible for nucleotide binding.^5^ The active site of these proteins contain a phosphate-binding loop (or P-loop) required for nucleotide and Mg^2+^ binding and to prevent spontaneous exchange between GDP and GTP.^6^ Conformational changes occur when the protein is bound to either GTP or GDP molecules, leading to structural rearrangements on two regions of the G domain (the switch regions) that determine the interaction of the GTPase with other proteins (effector proteins).^7,8^

Deactivation of Ras proteins or “turning off” the molecular switch requires GTP hydrolysis, resulting in the formation of GDP and inorganic phosphate (P_i_). This reaction is slow in solution and needs to be catalyzed. To reach full GTPase activity, the GTPase activating proteins (GAPs) must be recruited to accelerate hydrolysis by a factor of 10^5^ to 10^6^ fold. Figure 1 shows the X-ray structure of the Ras homologue family member A (RhoA) activated by a GAP (p50RhoGAP protein), with GTP and Mg^2+^ in the active site (PDB code 1OW3). GAP proteins contain a crucial arginine residue, known as the “arginine finger” that, after binding to the Ras-like protein, is inserted into the GTP-binding site to facilitate GTP hydrolysis, as seen in Figure 1.^8,9^ This arginine residue establishes hydrogen bond interactions with the phosphoryl groups of GTP and stabilizes the negative charge developed on the β,γ-bridging-oxygen atom (O3β), which makes the γ-phosphate group (P_γ_) a better leaving group.^9–11^

**Figure 1:**
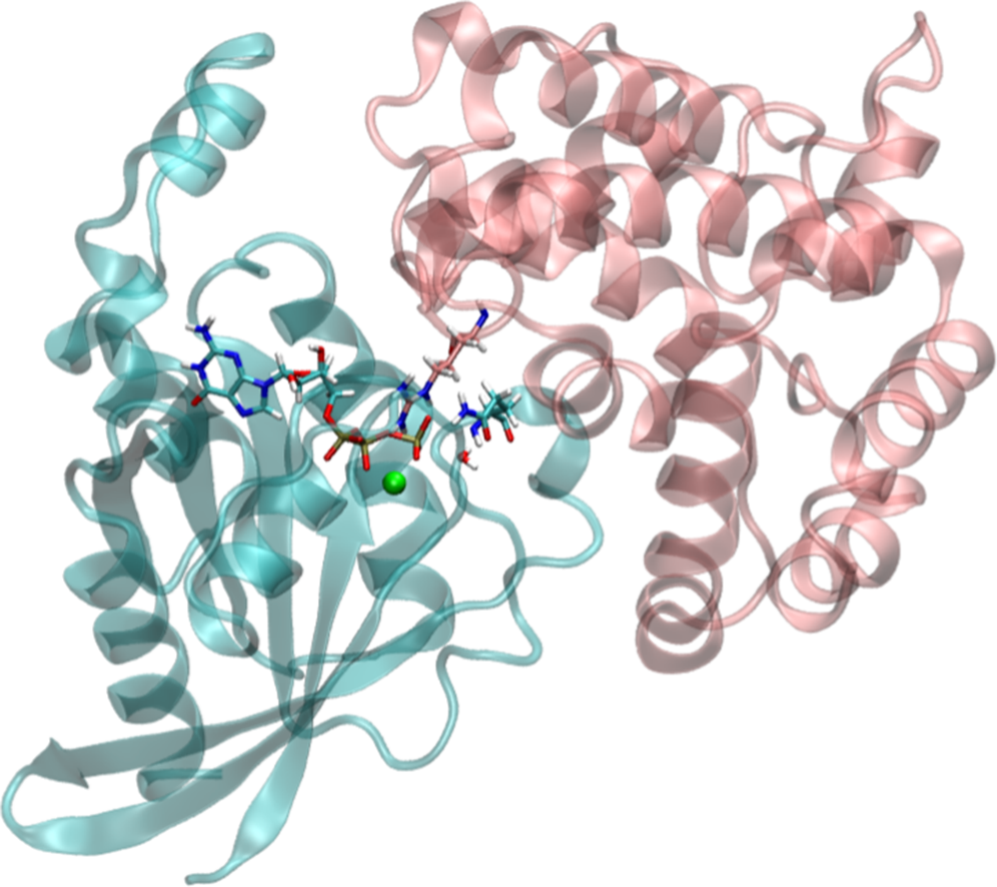
RhoA protein (in blue) in complex with p50RhoGAP (in pink). The GAP arginine finger (Arg85), Gln63, the nucleophilic water, and the GTP molecule are shown in a licorice representation, while the Mg ion is depicted as a green sphere.

RhoA, one of the most important members of the Ras family, has been demonstrated to regulate actin-dependent processes such as cell motility or cytokinesis, playing a key role in contractile ring formation.^12–16^ Additionally, RhoA is crucial for endothelial barrier function and has been related to several diseases, including breast, colon, or lung cancers.^6,15–17^ This has led to the emerging design of therapeutic agents targeting RhoA.^6^ However, for a rational design of these agents, it is necessary to fully understand the RhoA molecular switch mechanism, including GTP hydrolysis. GTP hydrolysis by RhoA protein itself is slow, with a rate constant of 0.022 min^–1^ at 293 K. However, RhoA GTP hydrolysis can be enhanced by at least 4000-fold by activation with p50RhoGAP, resulting in a hydrolysis rate constant of 59.6 min^–1^,^12^ which corresponds to a free energy barrier of 17.2 kcal mol^–1^.

The important role of these proteins and the increasing number of crystal structures available have led to several studies trying to understand the GTP hydrolysis mechanism.^18,19^ Although many proposals have emerged, the catalytic mechanism remains controversial and has not yet been fully clarified. Previous computational studies were carried out using various methodologies, including empirical valence bond (EVB) simulations,^18,20,21^ ab initio molecular dynamics (MD),^4^ semiempirical quantum mechanics/molecular mechanics (QM/MM) free energy calculations,^22^ or density functional theory (DFT)/MM energy optimizations.^23^ The availability of low-lying d-orbitals on the phosphorus atom enables various possibilities for transition states (TSs) in GTP hydrolysis, with both associative and dissociative pathways being possible. In the associative mechanism, the nucleophilic attack occurs before the departure of the GDP leaving group, resulting in the formation of a pentacoordinated P_γ_ group, while in the dissociative pathway, the GDP leaves before the nucleophilic attack, forming a metaphosphate group. The dissociative pathway appears to be favored as the accumulation of negative charge on oxygen atoms can be stabilized inside a positively charged GTP-binding pocket. Besides, the nature of GDP as a good leaving group supports this preference.^3,10,19,20^ The mechanism for the activation of the nucleophilic water is also a subject of debate, with several proposed options illustrated in Scheme 1. In mechanism A (or the solvent-assisted pathway,) the nucleophilic attack of the water molecule leads to a short-lived intermediate that tautomerizes to form the H_2_PO_4_^–^ ion and a GDP moiety.^18,20^ In mechanism B (substrate-assisted mechanism), the nucleophilic water is activated by means of a proton transfer to the phosphate group that proceeds in a partially concerted fashion with the nucleophilic attack in a TS.^18,20,24^ Another variant is mechanism C, which involves the participation of two water molecules, with one water molecule providing a proton to the phosphate group while or after another water molecule performs the nucleophilic attack.^21,22^ Other studies have explored the potential role of a glutamine residue as a general base (Gln63/Gln61 in RhoA and Ras proteins, respectively) deprotonating the nucleophilic water molecule prior to the nucleophilic attack (mechanism D).^25^ However, subsequent research into this mechanism has dismissed the possibility of this residue acting as a base.^10,19,20,26^ Finally, given the confirmed importance of the glutamine residue in the reaction mechanism,^22,27^ an alternative pathway has been proposed (mechanism E), in which this residue mediates in the nucleophilic attack thanks to the amide–imide tautomerism.^21,23,28^ In this mechanism, the carbonyl group of a nearby glutamine residue abstracts a proton from the nucleophilic water molecule while the side chain nitrogen atom donates a proton to one of the nonbridging oxygen atoms of the phosphate group, causing the transformation of the glutamine from an amide to an imide form.

**Scheme 1:**
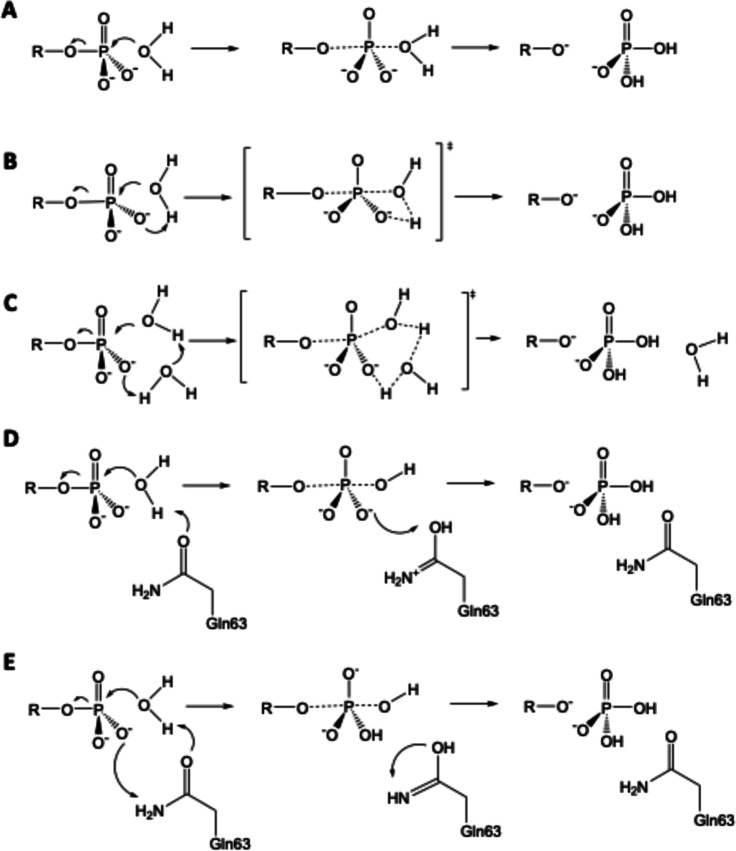
Mechanistic Proposals for GTP Hydrolysis in the Ras Superfamily; (A) Solvent-Assisted Mechanism; (B) Substrate-Assisted Mechanism; (C) Two-Waters Mechanisms; (D) Glutamine Base-Assisted Mechanism; and (E) Amide–Imide Mechanism

In this work, we present a QM/MM study of the GTP hydrolysis reaction mechanism in RhoA activated by p50RhoGAP. We have investigated the different mechanistic proposals discussed above, calculating their reaction free energy profiles along multidimensional free energy landscapes. The only mechanism displaying an activation free energy compatible with the experimental rate constant is the one involving the amide–imide tautomerism of Gln63, a residue present in the active site whose reactive orientation is fixed by means of a hydrogen bond established with a p50RhoGAP residue.

## 2 Methods

### 2.1 Molecular Dynamics Simulations

The Protein Data Bank structure with code 1OW3 was employed as a starting point in our study. This structure contains RhoA protein in complex with p50RhoGAP together with GDP-MgF_3_ (as a GTP analogue) and a Mg^2+^ ion in the active site.^29^ In order to prepare the simulations, the MgF_3_ moiety of the substrate was replaced with a phosphoryl group (P_γ_), thus obtaining GTP as the substrate. To perform this replacement, the UFSC Chimera software was employed,^30^ taking care of preserving the Mg^2+^ ion coordination with the added phosphoryl group. The missing hydrogen atoms were added to the system using the tleap tool of the Ambertools22 package.^31^ Protonation states at neutral pH were assigned using PropKa 3.5.1.^32,33^ Parameters for the GTP substrate were obtained from the work of Meagher et al.,^34^ complemented with the generalized amber force field.^35^ The partial charges were calculated by employing the restrained electrostatic potential method,^36^ at the HF/6-31G* level, as implemented in Gaussian16.^37^ Protein amino acids were described using the ff14SB force-field.^38^ Then, using the tleap tool, the p50RhoGAP-RhoA-GTP system was solvated in a box of water, making sure that the protein–substrate atoms were at least 10 Å from the limits of the simulation box. The water box was modeled at the TIP3P level.^39^ The charge of the system was neutralized by the addition of Na^+^ ions. MD simulations were performed with Amber 18,^40^ using periodic boundary conditions. The setup of the system followed a standard procedure consisting of four stages: minimization, heating, equilibration, and production. For minimization, four cycles of 4000 steps each were performed using constant volume. The force constant restraint of 200 kcal mol^–1^ Å^–2^ was applied to different atoms in each cycle to gradually minimize the system: in the first cycle, all atoms except hydrogens were restrained, in the second one, only the water molecules were nonrestrained, and in the third cycle, side chains were also released to finally release the backbone atoms, having a nonrestricted system in the last cycle. The first 200 steps of each cycle were calculated using the steepest descent method, and then, the algorithm was switched to the conjugated gradient method. Electrostatic interactions were calculated employing the particle-mesh Ewald method,^41,42^ and the cutoff range for short-ranged interactions was set at 10 Å. The cycles ended when the root-mean-square gradient was 10^–4^ kcal/mol. The minimized structures were heated from 0 to 300 K using a linear heating ramp and the Langevin dynamics thermostat, with the collision frequency set at 5.0 ps^–1^. Heating was carried out under constant pressure, using a Berendsen barostat (reference pressure of 1 atm), with a time step of 1 fs. During the heating, a light restraining parabolic potential was applied to the substrate and protein backbone atoms, setting a force constant of 20 kcal mol^–1^ Å^–2^. The configuration obtained after heating was equilibrated gradually, releasing the restraint at a rate of 2.4 kcal mol^–1^ Å^–2^ ns^–1^. Simulation parameters and the thermostat were the same as in the heating step. Once all the restraints were completely released, a production simulation of 500 ns in the *NVT* ensemble was run for 3 replicas (1.5 μs in total). We ran an additional 500 ns replica changing the solvent model to TIP4P^39^ to test the possible influence on the presence of water molecules in the active site. The production step was performed using the Amber18 GPU version of pmemd^43–45^ with a time step of 2 fs since the SHAKE algorithm^46^ was used to treat bonds involving hydrogen atoms.

### 2.2 QM/MM Calculations

Hybrid QM/MM simulations were performed to describe GTP hydrolysis reaction mechanisms in the RhoA/p50RhoGAP system. Two different levels of theory were used to describe the QM part to speed up the calculations. First, the different mechanisms proposed were studied at DFTB3/ff14SB.^38,47^ This method, based on tight binding with OPhyd^48^ specific parameters for phosphate hydrolysis reactions, allowed us to obtain a good approximation at a reasonable computational cost. Then, the most favorable mechanism was studied at the B3LYPD3/6-31G­(d)/ff14SB level,^38,49–53^ starting from the converged DFTB3 path. This methodology was already used in previous studies, yielding accurate results for ATP hydrolysis.^54^ The use of a high-level DFT method, free from system-specific parameters and widely recognized in the computational chemistry community, further reinforces the robustness of our conclusions.

The QM region in the DFTB3 studies contained the whole GTP substrate, the water molecule(s) implied in the hydrolysis, and the Mg^2+^ ion with its whole coordination sphere, comprising two water molecules and the side chains of Thr19 and Thr37. Due to its catalytic impact, the side chain of the Gln63 residue was also introduced in the QM subsystem as well as the side chains of the arginine finger, GAP-Arg85, and Lys18, which bear a positive charge that might polarize the substrate. The total QM region contained 120–123 atoms (depending on the reaction mechanism under consideration, see below) and had a neutral net charge. For the B3LYP-D3 calculations, to compensate the higher computational cost, the QM part was reduced to the phosphate groups of GTP, the side chain of the Gln63 residue, the nucleophilic water molecule, and the Mg^2+^ ion with its whole coordination sphere (see Figure S1). The QM/MM boundary was placed, when needed, between Cα and Cβ atoms, using the link-atom approach.^55,56^

### 2.3 Free Energy Calculations

GTP hydrolysis within the active site of the p50RhoGAP–RhoA complex is a process that involves changes in many degrees of freedom since several bonds must be formed and/or broken. The method employed in this work was the adaptive string method (ASM).^57^ This method allows us to obtain the minimum free energy path (MFEP) on the free energy surface (FES) of a process without lowering down its multidimensional nature while keeping it at a feasible computational cost. The FES is spanned in a space defined by the collective variables (CVs), which are the most important degrees of freedom related to GTP hydrolysis. Initially, N replicas of the system (the string nodes) are placed at different values of the CVs following an initial guess for the reaction path. Then, the position of the nodes evolves according to the free energy gradient, keeping them equidistant until the string reaches the MFEP. Finally, a single path-CV, denoted as s, that defines the position of the system along the MFEP is used to calculate the free energy profile.^58^

As in previous studies about enzymatic ATP hydrolysis,^54^ the set of CVs includes the distances associated with the bonds being broken and/or formed during the process and the pyramidalization coordinate of the P_γ_ atom. The choice of CV for each mechanism is detailed in the next section. Reaction mechanisms were explored using 92 to 144 nodes, depending on their complexity. The mass of the hydrogen atoms involved in proton transfers was changed to that of the deuterium to enhance convergence rate and sampling, allowing the use of a time step of 1 fs. The convergence of the ASM calculation was determined when the RMSD of the CVs was kept below 0.1 amu^1/2^·Å for at least 2 ps. Then, the simulations entered the umbrella sampling stage to obtain the conformational sampling along the path-CV. The amount of sampling at this stage was about 90 ps for the DFTB3 profiles and 10 ps for the B3LYPD3 one. The free energy profile was then computed using the weighted histogram analysis method.^59,60^

## 3 Results

In this section, we first discuss the results of the classical force field (MM) MD simulations of the enzyme–substrate complex (RhoA-p50RhoGAP containing GTP and a Mg^2+^ ion in the active site), and then the different reaction mechanisms investigated using QM/MM free energy calculations.

### 3.1 Enzyme–Substrate Complex

The MD simulation replicas were analyzed to evaluate the key protein–substrate interactions and the accessibility of the water molecules in the active site and its possible participation in the reaction mechanism. The time evolution of the RMSD with respect to the initial structure (see Figure S2) demonstrates the stability of the RhoA-p50RhoGAP-GTP complex prepared from the X-ray structure 1OW3. For all replicas, the RMSDs displayed small values, showing that there were no important conformational changes with respect to the X-ray structure during the classical MD simulations.

Analysis of the MD simulations of the enzyme–substrate complex highlighted some key interactions between RhoA protein, p50RhoGAP, and the Mg^2+^ ion and the GTP molecule to keep it in the active site, oriented for a nucleophilic attack (see Figure 2). The GTP molecule is attached to the RhoA protein through interactions with the phosphate-binding loop or P-loop. Specifically, four hydrogen bonds were found in all MD simulations in this region: Cys20 side chain polar hydrogen with the O1α of GTP (1.89 ± 0.2 Å), Lys18 with both the O1β (2.06 ± 0.2 Å) and the O2γ (1.89 ± 0.4 Å), and also the NH main chain group of Gly17 with the O1β (2.02 ± 0.5 Å). Gln63 also makes a strong hydrogen bond between one of the amide hydrogen atoms and GTP atom O1γ (1.85 ± 0.1 Å). Besides, Mg^2+^ has a key role in retaining the GTP molecule with interactions with O2β (1.92 ± 0.04 Å) and O3γ (1.87 ± 0.03 Å). The activating protein p50RhoGAP also establishes hydrogen bonding interactions with the phosphoryl groups of GTP. In particular, the arginine finger (GAP-Arg85) interacts with O2α (1.86 ± 0.1 Å), the β,γ-bridging-oxygen atom O3β (2.33 ± 0.2 Å), and the γ-phosphate group. For this last group, the two interactions of the arginine finger, with O1γ (2.01 ± 0.2 Å) and O3γ (2.87 ± 0.2 Å), are important to orient it for the nucleophilic attack.

**Figure 2:**
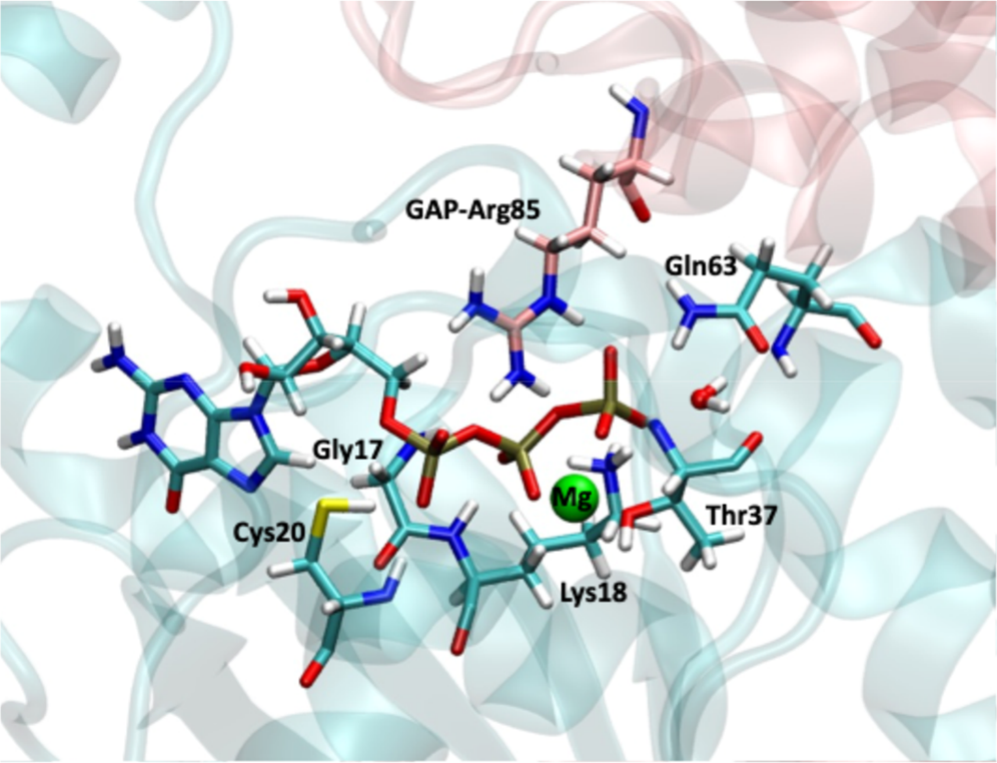
Interactions established between GTP and the nucleophilic water molecule with protein residues of the RhoA-p50RhoGAP complex.

Another significant piece of information obtained from the MD simulations was the presence of water molecule(s) in the active site of the RhoA-p50RhoGAP Michaelis complex. To analyze this issue, we computed the radial distribution function (RDF) of the water molecule’s oxygen atoms around the GTP P_γ_ and the Gln63 carbonyl oxygen atoms. The integrated RDFs obtained for the three replicas where we used the TIP3P force field for water and the additional replica carried out with the TIP4P force field are shown as Figure S3. The integration of the RDF gives the average number of water molecules found up to a given distance from the reference atom. The results are consistent among replicas and models with minor statistical differences. According to the simulations, there are three water molecules at distances between 3 and 5 Å of the GTP P_γ_ atom. Two of these molecules correspond to the water molecules coordinated to the magnesium ion. In addition, a third water molecule is observed within this range of distances, corresponding to nucleophilic water. The average distance between the oxygen atom of the nucleophilic water molecule and P_γ_ was 4.27 ± 1.2 Å. This water molecule is found to provide a hydrogen bond to the side chain carbonyl group of Gln63, with an average distance of 2.81 ± 0.7 Å. The nucleophilic water also receives a hydrogen bond from the Thr37 main chain NH group 2.63 ± 0.8 Å. A second water molecule in the second solvation sphere (around 6–6.5 Å from P_γ_) is found in at least 50% of the frames in the TIP3P and TIP4P simulations. Integrated RDFs around the Gln63 carbonyl oxygen atom in these simulations (Figure S3) confirm that there are 2 water molecules located between 3 and 6 Å away from this atom.

As discussed above, Gln63 plays a prominent role in theRhoA-p50RhoGAP complex with GTP. This residue establishes robust hydrogen bond interactions with both the γ-phosphate group of GTP and the nucleophilic water molecule, the two main reagents in the hydrolysis reaction. In this sense, p50RhGAP plays a decisive role in the process, contributing to the correct orientation of the Gln63 side chain in the active site, which in turn makes possible the interactions of this residue with GTP and the nucleophilic water. One of the hydrogen atoms of the Gln63 amide group (see Figure 2) forms a very stable hydrogen bond interaction with the main chain carbonyl group of GAP-Arg85, with an average distance of 1.90 ± 0.1 Å. The role of GAP would then not be limited to the presence of an arginine finger.

### 3.2 QM/MM Calculations on GTP Hydrolysis Mechanisms

We present here the results of our exploration of different possible reaction mechanisms for GTP hydrolysis in the active site of the RhoA-GAP complex. First, we employed DFTB3/MM simulations to evaluate the most plausible mechanism and/or discard other mechanisms proposed in the literature, based on their activation free energies. Note that not all of the variants presented in Scheme 1 were found during our free energy explorations. The most favorable reaction mechanism was then reevaluated using B3LYPD3/MM simulations. In all cases, we determined the MFEP using the ASM, as explained above.

#### 3.2.1 Solvent-Assisted Mechanism

The first mechanism explored was the solvent-assisted mechanism. The resulting DFTB3/MM free energy profile and evolution of the CVs that define this mechanism are shown in Figure 3. The first step in this mechanism is the dissociation of the O_β_–P_γ_ bond (CV1), leading to the formation of a planar meta-phosphate group that approaches the nucleophilic water, forming a first shallow intermediate (I1). This is reflected in the crossing of the O_β_–P_γ_ (CV1) and O_W_–P_γ_ (CV2) distances at ∼2.5 Å and in the hybridization coordinate of the γ-phosphate group (that measures deviation from planarity) that takes a value of ∼0 Å (CV6 in Figure 3b). The meta-phosphate state is reached through a low activation free energy of 4.9 kcal mol^–1^, corresponding to TS1 (see Figure 3d) This process is favored since the charge transfer to O_β_ is stabilized by electrostatic interactions with surrounding residues, such as GAP-Arg85 and Lys18.^4,10,61^ After this intermediate, the nucleophilic attack of the water molecule takes place, as seen by the evolution of the CV2 distance, which leads to a second TS (see TS2, in Figure 3e) and then to another short-lived intermediate (I2). This process does not require a high free energy activation since it is assisted by both Gln63 and Thr37 carbonyl groups, which are oriented toward the hydrogen atoms of the nucleophilic water molecule, polarizing the water molecule and enhancing its nucleophilicity.^4,11,23^ To complete the formation of H_2_PO_4_^–^, this intermediate must suffer a tautomerization process, during which a proton initially bonded to the water oxygen (O_W_) must be transferred to one of the nonbridging oxygen atoms bonded to P_γ_. In our case, the only viable mechanism found for this tautomerization was a direct proton transfer from the O_W_ to the O1γ. This proton transfer is reflected in the crossing of the H_W_–O1γ and H_W_–O_W_ distances (CV3 and CV4, respectively) at ∼1.2 Å. This proton transfer determines the third TS (TS3 in Figure 3f). Thus, in this case, the “solvent-assisted” and “substrate-assisted” mechanisms almost converge, the only difference being the existence of the shallow intermediate I2 in the former. The TS for this proton transfer (TS3) is the rate-limiting step of the reaction, with an activation free energy of 27.4 ± 0.2 kcal mol^–1^, in contrast with previous studies^20^ that suggested that this tautomerization could be a fast process and not rate-limiting. The high barrier for this step can be explained by the formation of a strained four-membered ring at TS3 and the existence of electrostatic interactions between GAP-Arg85 and Gln63 residues with the O1γ atom that decrease its basicity. As discussed below, this could be alleviated by the participation of another water molecule.

**Figure 3:**
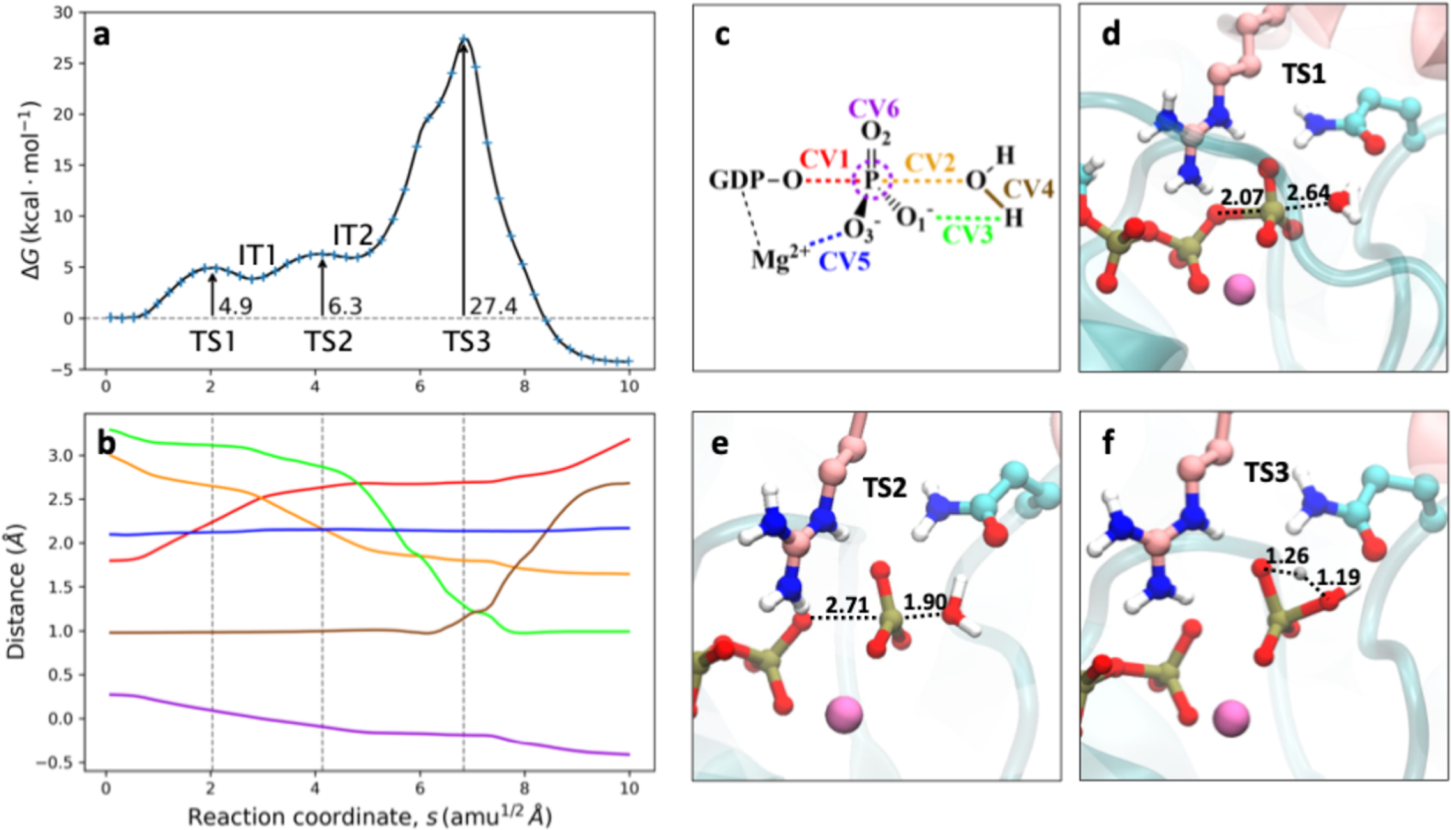
Solvent-assisted mechanism for the GTP hydrolysis in RhoA-p50RhoGAP. (a) DFTB3/MM free energy profile along the path-CV (s) for GTP hydrolysis. (b) Evolution of the CVs along the path-CV. The color code corresponds to their definition in panel c. (c) Definition of the CVs employed for the solvent-assisted reaction mechanism: distances between O_β_–P_γ_ (CV1) and O_W_–P_γ_ (CV2) for the nucleophilic displacement, distances between O1γ–H_W_ (CV3) and O_W_–H_W_ (CV4) for the proton transfer to form H_2_PO_4_^–^ and O_3_–Mg^2+^ (CV5) for the coordination of γ-phosphate to the divalent ion. The plane inversion of the P_γ_ atom (CV6) is measured as the distance between P_γ_ and the plane defined by the three bonded oxygen atoms. (d–f) Snapshots corresponding to TS1, TS2, and TS3, respectively (distances, corresponding to the coordinates of the MFEP, are given in Å).

With the results obtained for this mechanism, we confirmed that the first part of the mechanism, metaphosphate formation and nucleophilic attack, presents a low activation free energy barrier. However, the rate-limiting step (which corresponds to the proton transfer from the nucleophilic water molecule to the phosphate group) has an activation free energy of 27.4 ± 0.2 kcal mol^–1^, about 10 kcal·mol^–1^ above the experimental estimation. Previous studies^4^ have already evidenced the difficulties of a direct proton transfer between the nucleophilic water and the phosphate group. In order to investigate whether the tautomerization process could be favored by the presence of a second water molecule that reduces the strain at TS3, we performed new QM/MM simulations including an additional water molecule in the reacting region.

#### 3.2.2 Two Water Molecules Mechanism

Previous studies on GTP hydrolysis in solution,^21^ and in Ras proteins without GAP,^22^ have shown that the inclusion of a second water molecule in the active site could assist the proton transfer step, reducing the activation free energy of the process. For the study of the mechanism of two water molecules (see Figure 4), we selected an active site configuration displaying two water molecules around the γ-phosphate group of GTP, about 3 Å away from each other, in agreement with the RDF analysis discussed above (see Figure 4d), and both were included in the QM region. The first part of the mechanism proceeds as in the solvent-assisted mechanism, with an initial O_β_–P_γ_ bond cleavage (TS1) forming the metaphosphate structure that is now hydrogen bonded (1.8 Å) to the second water molecule and then the nucleophilic attack of the water molecule (TS2). In this initial part of the mechanism, the free energy activation is now 16.5 kcal mol^–1^. The barrier is higher than that obtained for the solvent-assisted free energy landscape (Figure 3). The increment can be explained by the steric hindrance of two water molecules in a reduced space, resulting in a slightly larger distance from the nucleophilic water to the P_γ_ atom (compare CV2 initial values in Figures 3b and 4b) and the necessary reorientation of the nucleophilic water molecule to attack P_γ_. Also, the presence of the second water molecule weakens the interactions of the metaphosphate group with Gln63 and GAP-Arg85 (Figure S4). Despite this increment, the observed free energy barrier until this point, 16.5 kcal mol^–1^, is plausible for an enzymatic process. However, the rate-limiting step of this two water reaction mechanism is still the proton transfer from the nucleophilic water molecule to the γ-phosphate group, now mediated by the second water molecule. As shown in TS3 in Figure 4e, this step corresponds to a concerted double proton transfer: one from the second water molecule to the O1γ (CV5 and CV6 in Figure 3b) and one from the nucleophilic water molecule to the water molecule that is donating its proton (CV7 and CV8, Figure 3b), leading to the formation of H_2_PO_4_^–^. The overall activation free energy for this reaction mechanism is 30.6 ± 0.3 kcal mol^–1^, and thus, this reaction mechanism was also discarded as a plausible one. While the inclusion of a second water molecule facilitates a less tensioned transition structure for the proton transfer from water to the γ-phosphate group, it increases the free energy cost of the first part of the mechanism: metaphosphate formation and nucleophilic attack, resulting in a global free energy barrier slightly higher than for the solvent-assisted mechanism.

**Figure 4:**
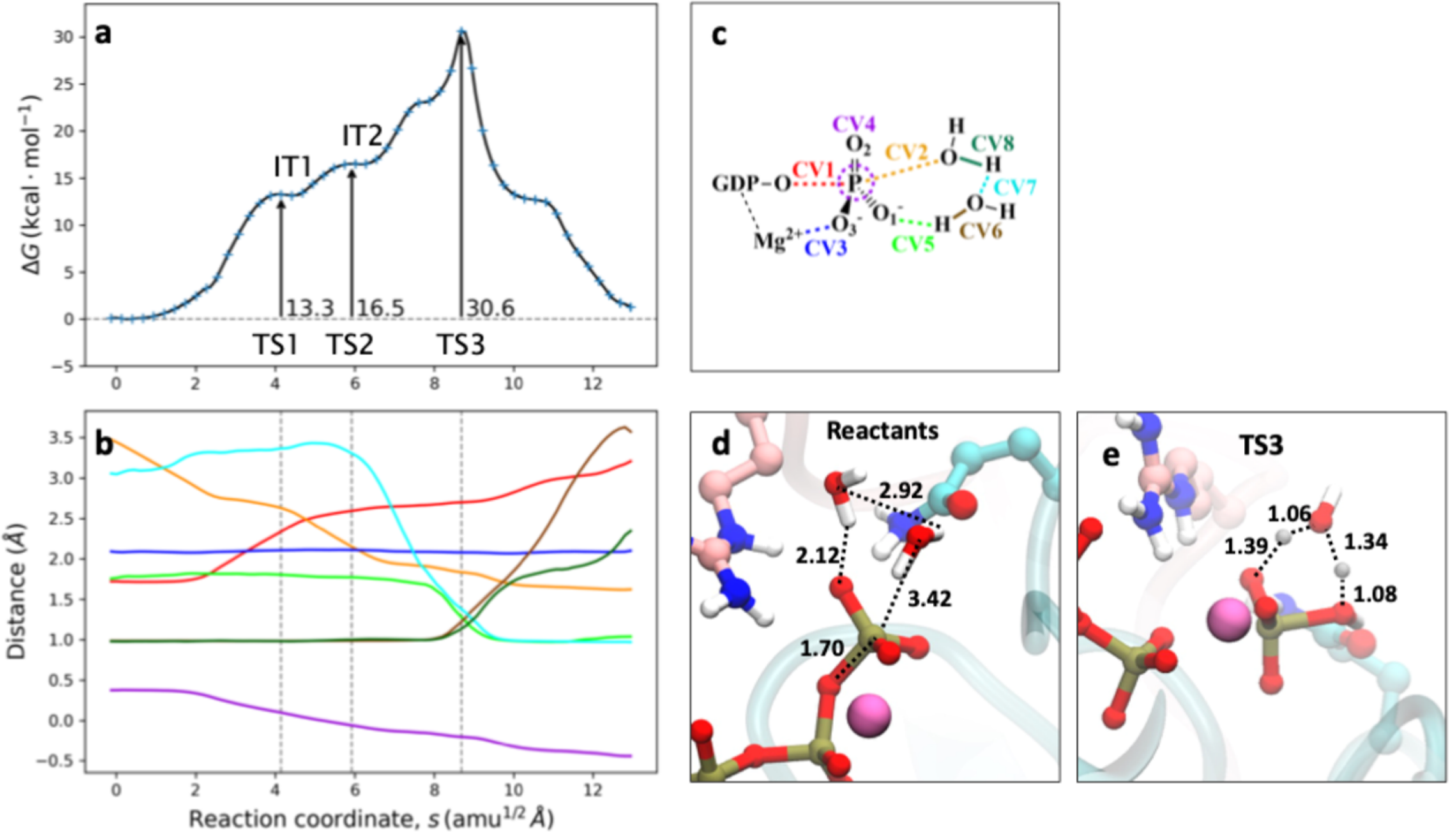
Two water molecules mechanism for the GTP hydrolysis in RhoA-p50RhoGAP. (a) DFTB3/MM free energy profile along the path-CV (s) for GTP hydrolysis. (b) Evolution of the CVs along the path-CV. The color code corresponds to their definition in panel c, (c) Definition of the CVs employed for the solvent-assisted reaction mechanism: distances between O_β_–P_γ_ (CV1) and O_W_–P_γ_ (CV2) for the nucleophilic displacement and distance O_3_–Mg^2+^ (CV3) for the coordination of γ-phosphate to the divalent ion. The plane inversion of the P_γ_ atom (CV4) is measured as the distance between P_γ_ and the plane defined by the three bonded oxygen atoms. Distances between protons and water molecule oxygen atoms (CV5, CV6, CV7, and CV8) define the proton transfer to the γ-phosphate group. (d,e) Snapshots corresponding to the reactant state and TS3, respectively (distances, corresponding to the coordinates of the MFEP, are given in Å).

#### 3.2.3 Amide–Imide Tautomerism Mechanism

In GTP hydrolysis catalyzed by Ras, the action of a glutamine residue in the active site has been demonstrated to be crucial, although its role has been a subject of debate. While its participation as a catalytic base was discarded, some studies proposed that its role is far more important, taking part in the proton transfer to the phosphate group.^4,21–23,27,28,62^ In the proposed glutamine mediated mechanism (Scheme 1), the nucleophilic water molecule donates a proton to the Gln63 carbonyl group, which in turn transfers the proton to the O1 atom of the γ-phosphate group, forming the H_2_PO_4_^–^ molecule. We did not find a reaction path corresponding to this description in our QM/MM calculations. Instead, we explored the possibility where Gln63 participates through an amide–imide tautomerism, where the amide group transfers a proton to the γ-phosphate group while the carbonyl group abstracts the proton from the nucleophilic water molecule. This represents the first part of the reaction as the second part involves the regeneration of the active site, the Gln63 tautomeric conversion from its imide form back to the amide form.

According to our DFTB3/MM exploration of the FES (see Figure 5), the first steps of this glutamine-mediated mechanism do not differ from the solvent-assisted mechanism, in such a way that the pathway also starts with a low barrier of the O_β_–P_γ_ bond dissociation (TS1), to form metaphosphate (IT1), as reflected by the crossing of the O_β_–P_γ_ (CV1) and the distances of the O_W_–P_γ_ (CV2) at ∼2.5 Å and in the hybridization coordinate of the γ-phosphate group (CV4) that takes a value of ∼0 Å (see Figure 5b,c). Then, the nucleophilic attack of the water molecule on the P_γ_ atom takes place. This attack is assisted by electrostatic interactions with Gln63 that polarizes the nucleophilic water molecule. Once the nucleophilic attack has started, a proton is transferred from the water molecule to the Gln63 carbonyl group, as deduced from the evolution of the O_W_–H_W_ (CV8) and H_W_–O_Gln_ (CV7) distances in Figure 5b, which leads to TS3, shown in Figure 5d. This step has an activation free energy of 7.1 kcal mol^–1^. This low barrier can be explained by the electronic delocalization in the amide group of Gln63 that favors an imide form of glutamine’s side chain, where the carbonyl oxygen atom becomes protonated. Electrostatic interactions within the active site also favor this proton transfer process. Glutamine basicity is enhanced by the hydrogen bond established by glutamine’s amide group with the carbonyl group of Arg85 and with the γ-phosphate group. In agreement with this observation, this first proton shift is rapidly compensated in TS4 (shown in Figure 5e), in which the proton is subsequently transferred from the Gln63 amide group to the O1 atom of the γ-phosphate group atom, with a free energy barrier of 6.9 kcal mol^–1^, resulting in the formation of the neutral imide form of Gln63 (IT4). This process is assisted by electrostatic interactions between the GAP arginine finger (Arg85) and the imidic nitrogen atom that donates the proton.

**Figure 5:**
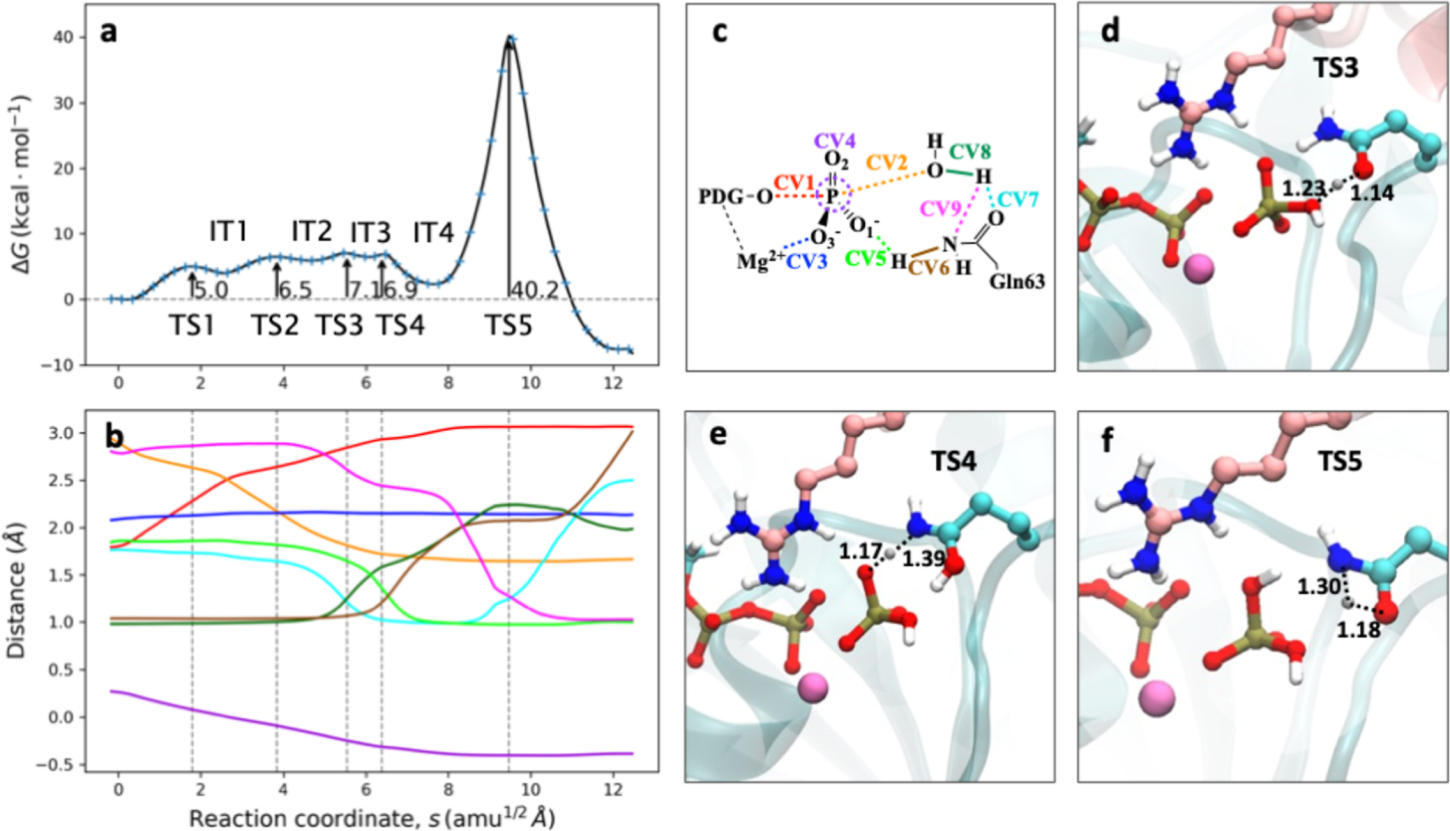
Amide–imide tautomerism reaction mechanism for the GTP hydrolysis in RhoA-p50RhoGAP. (a) DFTB3/MM free energy profile along the path-CV(s) for GTP hydrolysis. (b) Evolution of the CVs along the path-CV (left). The color code corresponds to the CVs definition in panel c. (c) Scheme of the CVs employed for the Gln-mediated reaction mechanism: distances between O_β_–P_γ_ (CV1) and O_W_–P_γ_ (CV2) for the cleavage of the phosphoanhydride bond and nucleophilic attack, O_3_–Mg^2+^ (CV3) for the coordination of γ-phosphate to the divalent ion and plane inversion of the P atom (CV4), measured as the distance between P_γ_ and the plane defined by the three bonded oxygen atoms. The proton transfer from the Gln63 amide to phosphate group to form H_2_PO_4_^–^ is described by O1γ–H_GLN_ (CV5) and N_Gln_–H_Gln_ (CV6), while proton transfer from the water molecule to Gln63 carbonyl group is described by O_Gln_–H_W_ (CV7) and H_W_–O_W_ (CV8). The intramolecular proton transfer for Gln regeneration is controlled by O_Gln_–H_W_ (CV7) and N_Gln_–H_W_ (CV9) distances. (d–f) Snapshots corresponding to TS3, TS4, and TS5, respectively (distances, corresponding to the coordinates of the MFEP, are given in Å).

Once Gln63 is in its imide form, the GTP hydrolysis reaction is completed, and H_2_PO_4_^–^ formation is achieved with a very low free energy barrier. However, the complete reaction pathway requires the regeneration of Gln63 to prepare the active site for the next turnover. Part two of the proposed mechanism, which consists of Gln63 regeneration, is not so favorable, as can be seen in the free energy profile of Figure 5a, where the last TS (TS5) is the rate-limiting step. In this mechanism, the tautomeric change back from the imide to the amide form takes place through a direct proton transfer from the O_Gln_ atom to the N_Gln_ atom, which has an associated free energy barrier of 40.2 ± 0.3 kcal mol^–1^, a value that is too high for a plausible enzymatic mechanism. At TS5, the distances of the O_Gln_–H and N_Gln_–H (represented by CV7 and CV9 in Figure 5b) bonds cross approximately at 1.2–1.3 Å. The resulting four-cycle TS structure is highly strained, as observed in Figure 5f. The rate-limiting step for this mechanism is then Gln63 regeneration, with a free energy barrier that discards this mechanism or at least its second part, the intramolecular regeneration of the Gln63 residue.

#### 3.2.4 Amide–Imide Mechanism with Phosphate-Assisted Gln Regeneration

Our next step, following the work of Khrenova et al.^23^ that studied the potential energy surface for GTP hydrolysis in the Ras-GAP complex, was to explore the possibility of glutamine regeneration mediated by the already formed H_2_PO_4_^–^ ion, acting as a proton shuttle between the nitrogen and oxygen atoms of the Gln63 side chain. The results of the free energy exploration at the DFTB3/MM level are listed in Figure 6.

**Figure 6:**
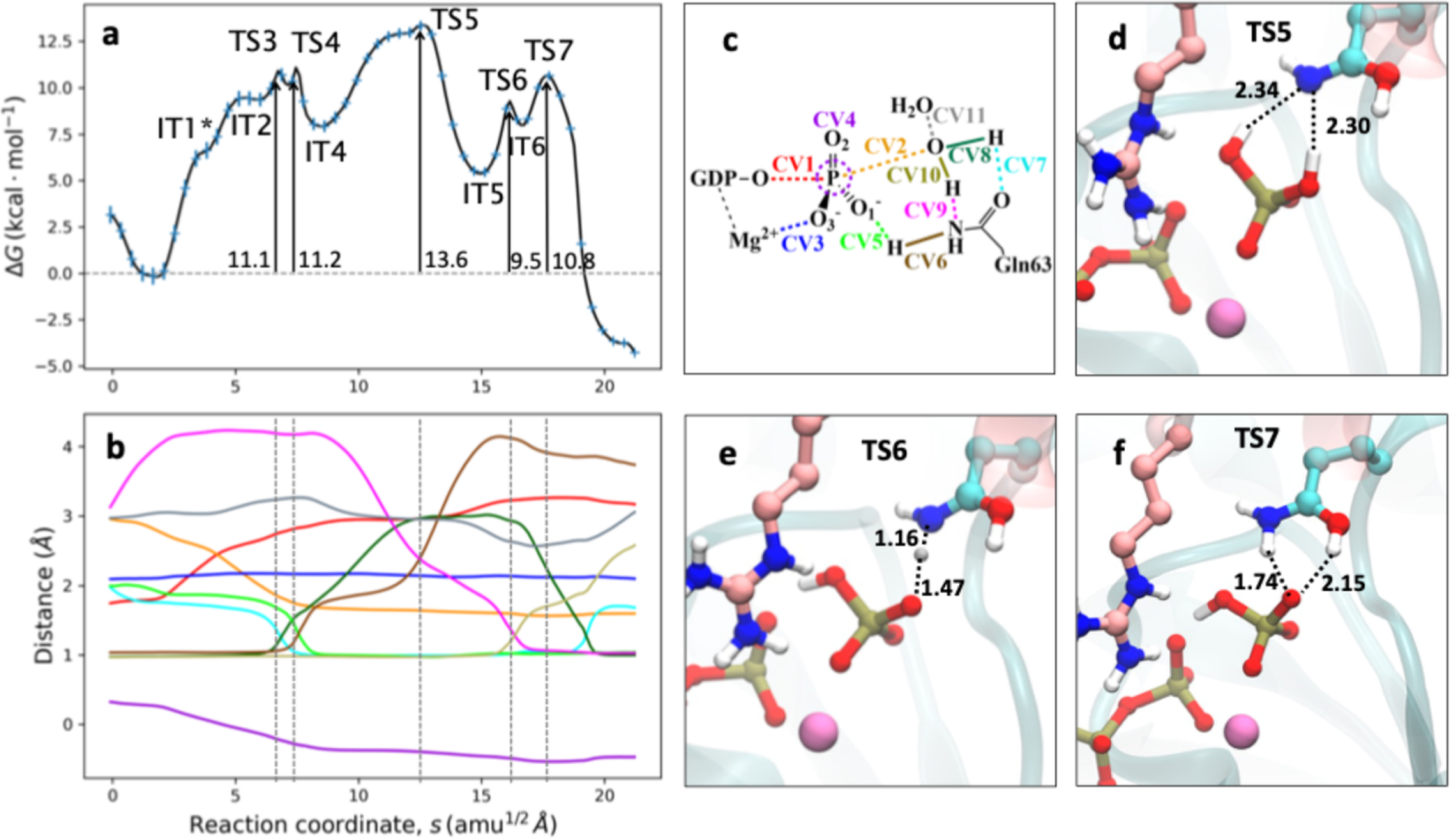
Amide–imide tautomerism reaction mechanism with assisted regeneration for the GTP hydrolysis in RhoA-p50RhoGAP. (a) DFTB3/MM free energy profile along the path-CV(s) for GTP hydrolysis. (b) Evolution of the CVs along the path-CV. The CVs changes are colored according to their definition shown in scheme c. (c) Scheme of the CVs employed for the reaction mechanism: distances between O_β_–P_γ_ (CV1) and O_W_–P_γ_ (CV2) for the cleavage of the phosphoanhydride bond and nucleophilic attack, O_3_–Mg^2+^ (CV3) for the coordination of γ-phosphate to the divalent ion, and plane inversion of the P atom (CV4), measured as the distance between P_γ_ and the plane defined by the three bonded oxygen atoms. The proton transfer from Gln63 to the γ-phosphate group to form H_2_PO_4_^–^ is described by O1γ–H_Gln_ (CV5) and N_Gln_–H_Gln_ (CV6), while the proton transfer from the phosphate group to Gln63 to tautomerize the residue is described by O_Gln_–H_W_ (CV7) and H_W_–O_W_ (CV8). These last two CVs also control Gln63 regeneration along with N_Gln_–H_W_ (CV9) and O_W_–H_W_ (CV10). A final distance between nucleophilic water and another water molecules (CV11) is introduced to check the possibility of another water molecule participating in the reaction mechanism. (d–f) Snapshots of TS5, TS6, and TS7, respectively (the most relevant distances in each case, corresponding to the coordinates of the MFEP, are given in Å).

The first part of the mechanism, until the formation of the inorganic phosphate, takes place as in the previous mechanism, displaying an activation free energy below 11 kcal·mol^–1^ (see Figure 6a, up to IT4). The only difference that can be remarked is that in this mechanism, the metaphosphate state does not appear as a shallow minimum along the free energy profile but as a shoulder (represented as IT1* in Figure 6a). After the nucleophilic attack of the water molecule on the metaphosphate group and the Gln-mediated proton transfer to the γ-phosphate group, the H_2_PO_4_^–^ ion is formed and Gln63 is present in its imide form (I4 in Figure 6a). In this structure, Gln63 is doubly hydrogen bonded to the inorganic phosphate, receiving a proton on the imidic nitrogen atom and donating a hydrogen from the hydroxyl group to the oxygen atom of the nucleophilic water, now bonded to the P_γ_ atom. The rate-limiting step of this mechanism is TS5 (see Figure 6d) that corresponds to a reorientation of the H_2_PO_4_^–^ ion, in agreement with previous studies on this mechanism.^23^ This step, with an activation free energy of 13.6 kcal·mol^–1^, is necessary for the inorganic phosphate to assist in Gln63 regeneration. This reorientation involves rotation around two P_γ_–O bonds, orienting the hydrogen atoms in such a way that the inorganic γ-phosphate now forms hydrogen bonds with the O3β atom of the β-phosphate group and with the nitrogen atom of Gln63 (intermediate IT5). Then, the tautomerization of the Gln residue back to the amide form is achieved through two proton transfers (see TS6 and TS7 in Figure 6e,f). In TS6, a proton is transferred from the inorganic phosphate to the Gln63 nitrogen atom, as reflected by the crossing of the N_Gln_–H_W_ (CV9) and H_W_–O_W_ (CV10) distances in Figure 6b. Then, in TS7, the phosphate group is reoriented to form a hydrogen bond interaction with the imide hydroxyl group. Finally, a proton is transferred from the imide hydroxyl group of Gln63 to the γ-phosphate group to restore again the H_2_PO_4_^–^ ion, involving changes in O_Gln_–H_W_ (CV7) and H_Gln_–O1γ (CV5) distances, and to recover the amide form of Gln63. This last proton transfer takes place downhill along the free energy profile as it involves a positively charge proton donor and a negatively charged proton acceptor. Note that the formed H_2_PO_4_^–^ ion is hydrogen bonded to the β-phosphate group, so a monohydrogen phosphate could be formed after proton transfer to GDP. The resulting mechanism, with a phosphate-assisted regeneration of the Gln63 residue, exhibited an overall activation free energy of 13.6 ± 0.2 kcal·mol^–1^. Therefore, this mechanistic proposal presents a lower activation free energy among all of the studied mechanisms, and the predicted value is compatible with the activation free energy derived from the experimental rate constant, 17.2 kcal mol^–1^. Thus, this mechanism was selected for ASM simulations using a B3LYPD3/MM Hamiltonian to corroborate the results at a more accurate computational level.

Figure 7 shows the evolution of the free energy profile and the CVs, together with some relevant TS configurations, obtained for the amide–imide tautomerism mechanism with assisted regeneration at the B3LYPD3/6-31G­(d)/MM level. The results obtained at this computational level are qualitatively close to those obtained at the DFTB3/MM level, which reassures the adequacy of the DFTB3 Hamiltonian (with specific parameters) to describe phosphate hydrolysis.^48,54^ The first stage of the mechanism, which corresponds to metaphosphate formation and nucleophilic attack, displays a quite similar structural evolution, as evidenced by the comparison of the CVs evolution in Figures 6b and 7b. Some small quantitative differences appear in the free energy profiles. The most noticeable change in the energetic description of the first stage of the reaction at the two levels is that some of the shallow minima and small barriers present in the DFTB3/MM profile disappear now in the B3LYPD3/MM simulations, and only one TS (TS4, Figure 7c) is needed to conclude the first stage. This TS corresponds to an almost concerted double proton transfer from the amide nitrogen atoms of Gln63 to the O1γ atom of the phosphate group (see the crossing of CV5 and CV6 in Figure 7b) and from the nucleophilic water molecule to the carbonyl oxygen atom of the Gln63 side chain (CV7 and CV8). The proton transfers from the nucleophilic water molecule to Gln63 and from this residue to the γ-phosphate group appeared in two different TSs at the DFTB3/MM level (TS3 and TS4 in Figure 6a), with activation free energies of 11.1 and 11.2 kcal mol^–1^, respectively, while they are almost completely concerted now (TS4 in Figure 7a), with a free energy barrier of 18.3 kcal·mol^–1^. The free energy of the intermediate obtained after TS4, where Gln63 is found in the imide form, is 14.0 kcal·mol^–1^ at the B3LYPD3/MM level, while the DFTB3/MM value was 7.9 kcal·mol^–1^. Despite these differences, the description of the first part of the mechanism is quite similar at both computational levels.

**Figure 7:**
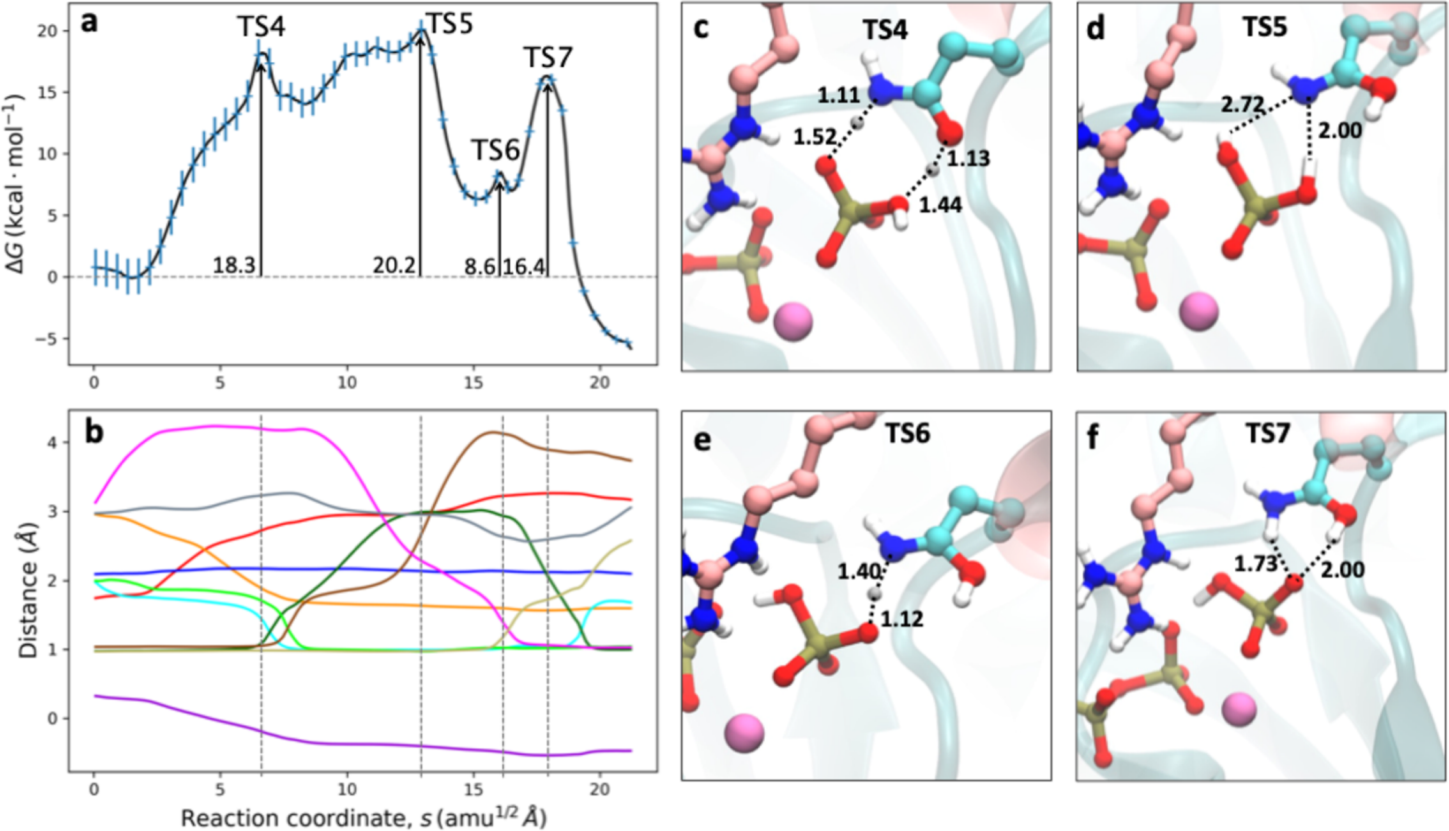
Amide–imide tautomerism reaction mechanism, with assisted Gln regeneration, for the GTP hydrolysis in RhoA-p50RhoGAP calculated at the B3LYPD3/MM level. (a) B3LYPD3/MM free energy profile along the path-CV(s) for GTP hydrolysis. (b) Evolution of the CVs along the path-CV. The CVs changes are colored according to their definition shown in panel 6c. (c–f) Snapshots of TS4, TS5, TS6, and TS7, respectively. (The most relevant distances in each case correspond to the coordinates of the MFEP and are given in Å).

The second part of the mechanism, regeneration of the amide form of Gln63, is also very similar at the two computational levels, and it is the rate-limiting step in both cases. Rearrangement of the hydrogen bonds established by the dihydrogen phosphate ion is also the rate-limiting step at the B3LYPD3/MM level, with an activation free energy of 20.2 ± 0.8 kcal·mol^–1^. The structure of TS5 is shown in Figure 6d. The two last TSs of the reaction (TS6 and TS7) correspond to the protons exchange between the phosphate group and Gln63 to restore the amide form of this residue. In TS6, a proton is transferred from the phosphate ion to the Gln63 imide nitrogen atom (Figure 7e). Finally, in TS7 (see Figure 7f), the phosphate group is reoriented to form a hydrogen bond with the imide hydroxyl group, from where the proton is transferred to form H_2_PO_4_^–^, recovering the amide form of the Gln63 side chain. This last proton transfer is exothermic.

At the B3LYPD3/MM level, the process is exergonic, with a reaction free energy of about −5.5 kcal·mol^–1^. The overall activation free energy obtained using B3LYPD3/MM simulations is determined by TS5, with a value of 20.2 ± 0.8 kcal·mol^–1^, in good agreement with the experimentally derived value of 17.2 kcal·mol^–1^. This agreement indicates that the proposed mechanism is plausible and a good candidate to explain the reactivity of this enzyme.

## 4 Conclusions

The Ras homologue family member A protein (or RhoA) is a member of the Ras superfamily of guanine nucleotide-binding proteins that is involved in several key cellular processes. These proteins act as molecular switches, cycling between the biologically active state and the biologically inactive state. The conformational transition between these two states is triggered by the hydrolysis of GTP, present in the active state, to produce GDP, present in the inactive state and inorganic phosphate. Despite the fundamental role of GTP hydrolysis in these enzymes, its mechanism is still a subject of debate. In this paper, we have conducted a systematic exploration of the reaction free energy landscape for GTP hydrolysis in RhoA, considering different mechanistic pathways previously proposed. Our approach is based on the exploration of a multidimensional free energy space with the ASM using validated QM/MM approaches and high-level DFT/MM simulations for the most suitable mechanistic candidate.

MD simulations of the RhoA-p50RhoGAP-GTP complex show some key interactions at the Michaelis complex. Residues Gly17, Lys18, and Cys20 of RhoA establish hydrogen bond interaction with the phosphate groups that together with the magnesium ion contribute to the binding of GTP. In addition, the GAP (p50RhoGAP) also makes important contributions to the binding of GTOP through the so-called arginine finger (GAP-Arg85). The guanidinium group of this residue makes hydrogen bond interactions with the three phosphate groups, mainly through atoms O2α, O3β, and O1γ. Apart from these interactions, the main chain carbonyl group of the arginine finger establishes a hydrogen bond interaction with the amide group of the Gln63 side chain, which contributes to place the side chain of this residue correctly oriented into the active site. Gln63 plays a fundamental role in GTP hydrolysis. First, this residue contributes, together with Thr37, to placing the nucleophilic water molecule close to the γ-phosphate group. Second, Gln63 is involved in the reaction mechanism.

All of the explored reaction mechanisms share some common steps. In all of them, GTP hydrolysis is initiated by the breaking of the phosphoanhydride bond between the β and γ phosphate groups to form metaphosphate. The approach of the nucleophilic water led to the formation of an adduct before the proton transfer events take places. These species are metastable and appear as very shallow minima or shoulders along the reaction free energy profiles. In the solvent-assisted mechanism, the subsequent proton rearrangements, required for the phosphate anion, are supposed not to be rate-limiting. However, our simulations show that the proton transfer from the nucleophilic water to the phosphate group, either directly or mediated by a second water molecule (two waters mechanism), requires overcoming a considerable free energy barrier, significantly higher than the experimentally derived value. Participation of Gln63 significantly reduces the energy cost associated with the proton rearrangement. The side chain carbonyl group of this residue can accept a proton from the nucleophilic water, while the amide group transfers a proton to the γ-phosphate, leading to the formation of an imide side chain. However, recovering the amide form of Gln63 now becomes the rate-limiting step. This final step is facilitated by a proton exchange between the imide group and the inorganic phosphate ion formed in the active site, with an activation free energy of 20.2 ± 0.8 kcal mol^–1^, which is in good agreement with the experimentally derived value of 17.2 kcal kcal mol^–1^. Beyond this agreement, the robustness of our conclusions regarding the most favorable mechanism for RhoA is supported by two additional factors. First, all mechanisms explored in this study were analyzed using the same computational setup, which minimizes the impact of uncertainties and ensures consistency in the comparison of relative barriers. Second, the free energy profile for the most favorable mechanism was recalculated by using a B3LYPD3/MM potential, a highly reliable method for this type of reaction, albeit at a significantly higher computational cost. Experimental testing of this mechanism could involve targeted mutations of the active site glutamine and/or its surrounding environment. FTIR spectroscopy has also been proposed as a valuable technique for detecting glutamine tautomerization as it could reveal the presence of tautomeric intermediates, such as those appearing after TS5 in Figure 7.^63–65^ Additionally, enzyme kinetic isotope effects^66^ could provide further insight into the validity of this mechanism, given that the rate-limiting TS involves the participation of glutamine.

The results of our DFT/MM exploration of the reaction free energy landscape in the active site of RhoA align with and confirm previous studies highlighting the critical role of glutamine amide–imide tautomerism in Ras enzymes,^23,28,63^ showing that this could be a general feature in the family. Indeed, amide–imide tautomerism involving glutamine and potentially asparagine may play a significant role in other enzymes by facilitating proton transfer events in complex reaction mechanisms. For instance, in glycosyltransferases,^67^ an active-site asparagine has been proposed to aid in proton shuttling. A pivotal role for asparagine or glutamine residues has also been described in a variety of enzymatic reactions, where their tautomerization may enhance nucleophilicity, such as during protein splicing,^68,69^ or enable them to act as bases in prenyltransferases^70^ and monoterpene synthases,^71^ and it could be also involved in the chain of reactions taking place after photoexcitation of flavin-binding protein domains.^63^ These examples illustrate the possible broad implications of glutamine and asparagine tautomerism in enzymatic mechanisms. In such cases, the recovery of the original amide form following tautomerization must also be considered a crucial step—potentially rate-limiting—in the overall reaction mechanism.

We conclude by emphasizing that while mechanisms involving amide–imide tautomerization may not initially appear to be the most favorable pathway, the electrostatic environment of highly charged enzymatic active sites, such as that of RhoA, can render this route accessible. Specific electrostatic interactions in enzymes are known to stabilize unexpected protonation states^72^ and facilitate nonintuitive proton transfer pathways,^73^ indicating that amide–imide tautomerization, and other processes, cannot be ruled out based solely on chemical intuition. In this context, QM/MM simulations have matured into powerful tools capable of accurately capturing the complex interplay of interactions within enzyme active sites and providing realistic mechanistic alternatives for enzymatic reactions. Their predictive capability is well established, as evidenced by studies demonstrating their ability to achieve chemical accuracy in enzyme mechanisms.^74^ Given the growing computational evidence supporting the role of amide–imide tautomerization in enzymatic catalysis, experimental validation is now essential to further confirm and explore this mechanism. We have pointed out several possibilities for this validation. We anticipate that a productive dialogue between experimental and computational approaches will be key to ultimately resolving this intriguing mechanistic possibility.

## Supplementary Material



## Data Availability

PDB files for the structures corresponding to the rate-limiting steps of each mechanism are available at: https://github.com/emedio/RhoA-p50RhoGAP.

## Notes

Figure with the definition of the QM region; additional details on system preparation; RMSD and RDF obtained during MD simulations; figure describing the interactions of the metaphosphate anion in the solvent-assisted and two-waters mechanisms, and description of the files provided in the repository (PDF)

## †

Instituto de Biocomputacion y Física de Sistemas Complejos (BIFI), Facultad de Ciencias, Universidad de Zaragoza, 50009 Zaragoza Spain

## References

[1] Wennerberg K., Rossman K. L., Der C. J. (2005). The Ras Superfamily at a Glance. J. Cell Sci..

[2] Colicelli J. (2004). Human RAS
Superfamily Proteins and Related GTPases. Sci
STKE.

[3] Rittinger K., Walker P. A., Eccleston J. F., Smerdon S. J., Gamblin S. J. (1997). Structure
at 1.65 Å of RhoA and Its GTPase-Activating Protein in Complex
with a Transition-State Analogue. Nature.

[4] Cavalli A., Carloni P. (2002). Enzymatic GTP Hydrolysis:
Insights from an Ab Initio
Molecular Dynamics Study. J. Am. Chem. Soc..

[5] Wittinghofer, A. Ras Superfamily Small G Proteins: Biology and Mechanisms 1 General Features, Signaling; Springer: Wien, 2014. 10.1007/978-3-7091-1806-1.

[6] Smithers C., Overduin M. (2016). Structural Mechanisms
and Drug Discovery Prospects
of Rho GTPases. Cells.

[7] Vetter, I. R. The Structure of the G Domain of the Ras Superfamily BT - Ras Superfamily Small G Proteins. In Biology and Mechanisms 1: General Features, Signaling; Wittinghofer, A. , Ed.; Springer Vienna: Vienna, 2014; pp 25–50. 10.1007/978-3-7091-1806-1_2.

[8] Zhang, S.-C. ; Nouri, K. ; Amin, E. ; Taha, M. S. ; Nakhaeizadeh, H. ; Nakhaei-Rad, S. ; Dvorsky, R. ; Ahmadian, M. R. Classical Rho Proteins: Biochemistry of Molecular Switch Function and Regulation BT - Ras Superfamily Small G Proteins. In Biology and Mechanisms 1: General Features, Signaling; Wittinghofer, A. , Ed.; Springer Vienna: Vienna, 2014; pp 327–340. 10.1007/978-3-7091-1806-1_14.

[9] Yi F., Kong R., Ren J., Zhu L., Lou J., Wu J. Y., Feng W. (2016). Noncanonical
Myo9b-RhoGAP Accelerates
RhoA GTP Hydrolysis by a Dual-Arginine-Finger Mechanism. J. Mol. Biol..

[10] Li G., Zhang X. C. (2004). GTP Hydrolysis Mechanism of Ras-like GTPases. J. Mol. Biol..

[11] Jin Y., Molt Jr. R. W., Waltho J. P., Richards N. G. J., Blackburn G. M. (2016). 19F NMR
and DFT Analysis Reveal Structural and Electronic Transition State
Features for RhoA-Catalyzed GTP Hydrolysis. Angew. Chem., Int. Ed..

[12] Zhang B., Zheng Y. (1998). Regulation of RhoA GTP Hydrolysis by the GTPase-Activating Proteins
P190, P50RhoGAP, Bcr, and 3BP-1. Biochemistry.

[13] Jordan S. N., Canman J. C. (2012). Rho GTPases in Animal
Cell Cytokinesis: An Occupation
by the One Percent. Cytoskeleton.

[14] Maddox A. S., Oegema K. (2003). Closing the GAP: A
Role for a RhoA GAP in Cytokinesis. Mol. Cell.

[15] Mosaddeghzadeh N., Ahmadian M. R. (2021). The RHO Family GTPases:
Mechanisms of Regulation and
Signaling. Cells.

[16] Lorenzano
Menna P., Cardama G. A., Comin M. J., Alonso D. F., Gómez D. E. (2010). Rho GTPasas Como Blancos Terapéuticos Relevantes
En Cáncer y Otras Enfermedades Humanas. Medicina.

[17] Chen S., Zhang Z., Zhang Y., Choi T., Zhao Y. (2022). Activation
Mechanism of RhoA Caused by Constitutively Activating Mutations G14V
and Q63L. Int. J. Mol. Sci..

[18] Calixto A. R., Moreira C., Kamerlin S. C. L. (2020). Recent Advances in Understanding
Biological GTP Hydrolysis through Molecular Simulation. ACS Omega.

[19] Carvalho A. T. P., Szeler K., Vavitsas K., Åqvist J., Kamerlin S. C. L. (2015). Modeling the Mechanisms of Biological GTP Hydrolysis. Arch. Biochem. Biophys..

[20] Calixto A. R., Moreira C., Pabis A., Kötting C., Gerwert K., Rudack T., Kamerlin S. C. L. (2019). GTP
Hydrolysis
Without an Active Site Base: A Unifying Mechanism for Ras and Related
GTPases. J. Am. Chem. Soc..

[21] Ram
Prasad B., Plotnikov N. V., Lameira J., Warshel A. (2013). Quantitative
Exploration of the Molecular Origin of the Activation of GTPase. Proc. Natl. Acad. Sci. U.S.A..

[22] Martín-García F., Mendieta-Moreno J. I., López-Viñas E., Gómez-Puertas P., Mendieta J. (2012). The Role of Gln61 in HRas GTP Hydrolysis: A Quantum
Mechanics/Molecular Mechanics Study. Biophys.
J..

[23] Khrenova M. G., Grigorenko B. L., Kolomeisky A. B., Nemukhin A. V. (2015). Hydrolysis of Guanosine
Triphosphate (GTP) by the Ras·GAP Protein Complex: Reaction Mechanism
and Kinetic Scheme. J. Phys. Chem. B.

[24] Molt R. W., Pellegrini E., Jin Y. (2019). A GAP-GTPase-GDP-Pi
Intermediate Crystal Structure Analyzed by DFT Shows GTP Hydrolysis
Involves Serial Proton Transfers. Chem. Eur.
J..

[25] Pai E. F., Krengel U., Petsko G. A., Goody R. S., Kabsch W., Wittinghofer A. (1990). Refined Crystal
Structure of the Triphosphate Conformation
of H-ras P21 at 1.35 A Resolution: Implications for the Mechanism
of GTP Hydrolysis. EMBO J..

[26] Sprang S. R. G. (1997). Protein
Mechanisms: Insights from Structural Analysis. Annu. Rev. Biochem..

[27] Schmidt G., Sehr P., Wilm M., Selzer J., Mann M., Aktories K. (1997). Gln 63 of Rho Is Deamidated
by Escherichia Coli Cytotoxic
Necrotizing Factor-1. Nature.

[28] Grigorenko B. L., Nemukhin A. V., Topol I. A., Cachau R. E., Burt S. K. (2005). QM/MM Modeling
the Ras–GAP Catalyzed Hydrolysis of Guanosine Triphosphate. Proteins: Struct., Funct., Bioinf..

[29] Graham D. L., Lowe P. N., Grime G. W., Marsh M., Rittinger K., Smerdon S. J., Gamblin S. J., Eccleston J. F. (2002). MgF3–
as a Transition State Analog of Phosphoryl Transfer. Chem. Biol..

[30] Pettersen E. F., Goddard T. D., Huang C. C., Couch G. S., Greenblatt D. M., Meng E. C., Ferrin T. E. (2004). UCSF Chimera—A
Visualization
System for Exploratory Research and Analysis. J. Comput. Chem..

[31] Case, D. A. ; Aktulga, H. M. ; Belfon, K. ; I. Y., Ben-Shalom , Berryman, J. T. ; Brozell, S. R. ; Cerutti, D. S. ; T. E., Cheatham, III , Cisneros, G. A. ; Cruzeiro, V. W. D. ; Darden, T. A. ; Duke, R. E. ; Giambasu, G. ; Gilson, M. K. ; H., Gohlke , Goetz, A. W. ; Harris, R. ; Izadi, S. ; Izmailov, S. A. ; Kasavajhala, K. ; Kaymak, M. C. ; King, E. ; Kovalenko, A. ; Kurtzman, T. ; Lee, T. S. ; LeGrand, S. ; Li, P. ; Lin, C. ; Liu, J. ; Luchko, T. ; Luo, R. ; Machado, M. ; V., Man , Manathunga, M. ; Merz, K. M. ; Miao, Y. ; Mikhailovskii, O. ; Monard, G. ; Nguyen, H. ; K. A., O’Hearn , A., Onufriev , Pan, F. ; Pantano, S. ; Qi, R. ; Rahnamoun, A. ; Roe, D. R. ; Roitberg, A. ; Sagui, C. ; Shajan, A. ; Shen, J. ; Simmerling, C. L. ; Skrynnikov, N. R. ; Smith, J. ; Swails, J. ; Walker, R. C. ; Wang, J. ; J., W ; Wei, H. ; Wolf, R. M. ; Wu, X. ; Xiong, Y. ; Xue, Y. ; York, D. M. ; Zhao, S. ; Kollman, P. A. . AMBER 22. University of California, San Francisco, 2022. https://ambermd.org/doc12/Amber22.pdf.

[32] Søndergaard C. R., Olsson M. H. M., Rostkowski M., Jensen J. H. (2011). Improved Treatment
of Ligands and Coupling Effects in Empirical Calculation and Rationalization
of PKa Values. J. Chem. Theory Comput..

[33] Olsson M. H. M., Søndergaard C. R., Rostkowski M., Jensen J. H. (2011). PROPKA3: Consistent Treatment of
Internal and Surface
Residues in Empirical PKa Predictions. J. Chem.
Theory Comput..

[34] Meagher K. L., Redman L. T., Carlson H. A. (2003). Development
of Polyphosphate Parameters
for Use with the AMBER Force Field. J. Comput.
Chem..

[35] Wang J., Wolf R. M., Caldwell J. W., Kollman P. A., Case D. A. (2004). Development
and Testing of a General Amber Force Field. J. Comput. Chem..

[36] Bayly C. I., Cieplak P., Cornell W., Kollman P. A. (1993). A Well-Behaved Electrostatic
Potential Based Method Using Charge Restraints for Deriving Atomic
Charges: The RESP Model. J. Phys. Chem..

[37] Frisch, M. J. ; Trucks, G. W. ; Schlegel, H. B. ; Scuseria, G. E. ; Robb, M. a. ; Cheeseman, J. R. ; Scalmani, G. ; Barone, V. ; Petersson, G. a. ; Nakatsuji, H. ; Li, X. ; Caricato, M. ; Marenich, a. V. ; Bloino, J. ; Janesko, B. G. ; Gomperts, R. ; Mennucci, B. ; Hratchian, H. P. ; Ortiz, J. V. ; Izmaylov, a. F. ; Sonnenberg, J. L. ; Williams; Ding, F. ; Lipparini, F. ; Egidi, F. ; Goings, J. ; Peng, B. ; Petrone, A. ; Henderson, T. ; Ranasinghe, D. ; Zakrzewski, V. G. ; Gao, J. ; Rega, N. ; Zheng, G. ; Liang, W. ; Hada, M. ; Ehara, M. ; Toyota, K. ; Fukuda, R. ; Hasegawa, J. ; Ishida, M. ; Nakajima, T. ; Honda, Y. ; Kitao, O. ; Nakai, H. ; Vreven, T. ; Throssell, K. ; Montgomery, Jr. J. a. ; Peralta, J. E. ; Ogliaro, F. ; Bearpark, M. J. ; Heyd, J. J. ; Brothers, E. N. ; Kudin, K. N. ; Staroverov, V. N. ; Keith, T. a. ; Kobayashi, R. ; Normand, J. ; Raghavachari, K. ; Rendell, a. P. ; Burant, J. C. ; Iyengar, S. S. ; Tomasi, J. ; Cossi, M. ; Millam, J. M. ; Klene, M. ; Adamo, C. ; Cammi, R. ; Ochterski, J. W. ; Martin, R. L. ; Morokuma, K. ; Farkas, O. ; Foresman, J. B. ; Fox, D. J. Gaussian 16, Revision C.01; Gaussian, Inc.: Wallin, 2016; .

[38] Maier J. A., Martinez C., Kasavajhala K., Wickstrom L., Hauser K. E., Simmerling C. (2015). Ff14SB: Improving
the Accuracy of
Protein Side Chain and Backbone Parameters from Ff99SB. J. Chem. Theory Comput..

[39] Jorgensen W. L., Chandrasekhar J., Madura J. D., Impey R. W., Klein M. L. (1983). Comparison
of Simple Potential Functions for Simulating Liquid Water. J. Chem. Phys..

[40] Case, D. A. ; I. Y., Ben-Shalom , Brozell, S. R. ; Cerutti, D. S. ; Cheatham, T. E., III , Cruzeiro, V. W. D. ; T. A., D ; Duke, R. E. ; Ghoreishi, D. ; Gilson, M. K. ; Gohlke, H. ; Goetz, A. W. ; Greene, D. ; Harris, R. ; Homeyer, N. ; Y, H. ; Izadi, S. ; Kovalenko, A. ; Kurtzman, T. ; Lee, T. S. ; LeGrand, S. ; Li, P. ; Lin, C. ; Liu, J. ; Luchko, T. ; Luo, R. ; D. J., Mermelstein , Merz, K. M. ; Miao, Y. ; Monard, G. ; Nguyen, C. ; Nguyen, H. ; Omelyan, I. ; Onufriev, A. ; Pan, F. ; R., Qi , Roe, D. R. ; Roitberg, A. ; Sagui, C. ; S., Schott-Verdugo , Shen, J. ; Simmerling, C. L. ; Smith, J. ; SalomonFerrer, R. ; Swails, J. ; Walker, R. C. ; Wang, J. ; Wei, H. ; Wolf, R. M. ; Wu, X. ; Xiao, L. , York, D. M. ; Zhao, S. ; Kollman, P. A. . AMBER 18: University of California, San Francisco, 2018. https://ambermd.org/doc12/Amber18.pdf.

[41] Darden T., York D., Pedersen L. (1993). Particle Mesh
Ewald: An N·log­(N)
Method for Ewald Sums in Large Systems. J. Chem.
Phys..

[42] Essmann U., Perera L., Berkowitz M. L., Darden T., Lee H., Pedersen L. G. (1995). A Smooth Particle
Mesh Ewald Method. J. Chem. Phys..

[43] Le
Grand S., Götz A. W., Walker R. C. (2013). SPFP: Speed without
Compromise—A Mixed Precision Model for GPU Accelerated Molecular
Dynamics Simulations. Comput. Phys. Commun..

[44] Salomon-Ferrer R., Götz A. W., Poole D., Le Grand S., Walker R. C. (2013). Routine
Microsecond Molecular Dynamics Simulations with AMBER on GPUs. 2.
Explicit Solvent Particle Mesh Ewald. J. Chem.
Theory Comput..

[45] Götz A. W., Williamson M. J., Xu D., Poole D., Le Grand S., Walker R. C. (2012). Routine Microsecond
Molecular Dynamics Simulations
with AMBER on GPUs. 1. Generalized Born. J.
Chem. Theory Comput..

[46] Ryckaert J.-P., Ciccotti G., Berendsen H. J. C. (1977). Numerical Integration of the Cartesian
Equations of Motion of a System with Constraints: Molecular Dynamics
of n-Alkanes. J. Comput. Phys..

[47] Gaus M., Cui Q., Elstner M. (2011). DFTB3: Extension
of the Self-Consistent-Charge Density-Functional
Tight-Binding Method (SCC-DFTB). J. Chem. Theory
Comput..

[48] Gaus M., Lu X., Elstner M., Cui Q. (2014). Parameterization of DFTB3/3OB for
Sulfur and Phosphorus for Chemical and Biological Applications. J. Chem. Theory Comput..

[49] Stephens P. J., Devlin F. J., Chabalowski C. F., Frisch M. J. (1994). Ab Initio Calculation
of Vibrational Absorption and Circular Dichroism Spectra Using Density
Functional Force Fields. J. Phys. Chem..

[50] Grimme S., Antony J., Ehrlich S., Krieg H. (2010). A Consistent and Accurate
Ab Initio Parametrization of Density Functional Dispersion Correction
(DFT-D) for the 94 Elements H-Pu. J. Chem. Phys..

[51] Becke A. D. (1993). A New Mixing
of Hartree–Fock and Local Density-functional Theories. J. Chem. Phys..

[52] Lee C., Yang W., Parr R. G. (1988). Development of the Colle-Salvetti
Correlation-Energy Formula into a Functional of the Electron Density. Phys. Rev. B.

[53] Vosko S. H., Wilk L., Nusair M. (1980). Accurate Spin-Dependent Electron
Liquid Correlation Energies for Local Spin Density Calculations: A
Critical Analysis. Can. J. Phys.

[54] García-Martínez A., Zinovjev K., Ruiz-Pernía J.
J., Tuñón I. (2023). Conformational
Changes and ATP Hydrolysis in Zika Helicase: The Molecular Basis of
a Biomolecular Motor Unveiled by Multiscale Simulations. J. Am. Chem. Soc..

[55] Field M. J., Bash P. A., Karplus M. (1990). A Combined Quantum Mechanical and
Molecular Mechanical Potential for Molecular Dynamics Simulations. J. Comput. Chem..

[56] Warshel A., Levitt M. (1976). Theoretical Studies of Enzymic Reactions: Dielectric,
Electrostatic and Steric Stabilization of the Carbonium Ion in the
Reaction of Lysozyme. J. Mol. Biol..

[57] Zinovjev K., Tuñón I. (2017). Adaptive Finite
Temperature String Method in Collective
Variables. J. Phys. Chem. A.

[58] Zinovjev K., Tuñón I. (2014). Exploring Chemical Reactivity of
Complex Systems with
Path-Based Coordinates: Role of the Distance Metric. J. Comput. Chem..

[59] Grossfield, A WHAM: the weighted histogram analysis method, version 2.0.9. http://membrane.urmc.rochester.edu/wordpress/?page_id=126 (accessed Jan 20, 2025).

[60] Kumar S., Rosenberg J. M., Bouzida D., Swendsen R. H., Kollman P. A. (1992). THE Weighted
Histogram Analysis Method for Free-Energy Calculations on Biomolecules.
I. The Method. J. Comput. Chem..

[61] Allin C., Gerwert K. (2001). Ras Catalyzes GTP Hydrolysis
by Shifting Negative Charges
from γ- to β-Phosphate As Revealed by Time-Resolved FTIR
Difference Spectroscopy. Biochemistry.

[62] Chen S., Zhang Z., Zhang Y., Choi T., Zhao Y. (2022). Activation
Mechanism of RhoA Caused by Constitutively Activating Mutations G14V
and Q63L. Int. J. Mol. Sci..

[63] Grigorenko B. L., Khrenova M. G., Nemukhin A. V. (2018). Amide–Imide Tautomerization
in the Glutamine Side Chain in Enzymatic and Photochemical Reactions
in Proteins. Phys. Chem. Chem. Phys..

[64] Domratcheva T., Hartmann E., Schlichting I., Kottke T. (2016). Evidence for Tautomerisation
of Glutamine in BLUF Blue Light Receptors by Vibrational Spectroscopy
and Computational Chemistry. Sci. Rep..

[65] Khrenova M. G., Grigorenko B. L., Nemukhin A. V. (2016). Theoretical Vibrational Spectroscopy
of Intermediates and the Reaction Mechanism of the Guanosine Triphosphate
Hydrolysis by the Protein Complex Ras-GAP. Spectrochim.
Acta, Part A.

[66] Silva R. G., Murkin A. S., Schramm V. L. (2011). Femtosecond
dynamics coupled to chemical
barrier crossing in a Born-Oppenheimer enzyme. Proc. Natl. Acad. Sci. U.S.A..

[67] Piniello B., Lira-Navarrete E., Takeuchi H., Takeuchi M., Haltiwanger R. S., Hurtado-Guerrero R., Rovira C. (2021). Asparagine Tautomerization in Glycosyltransferase
Catalysis. The Molecular Mechanism of Protein O-Fucosyltransferase
1. ACS Catal..

[68] Mujika J. I., Lopez X., Mulholland A. J. (2012). Mechanism
of C-Terminal Intein Cleavage
in Protein Splicing from QM/MM Molecular Dynamics Simulations. Org. Biomol. Chem..

[69] Barnard T. J., Gumbart J., Peterson J. H., Noinaj N., Easley N. C., Dautin N., Kuszak A. J., Tajkhorshid E., Bernstein H. D., Buchanan S. K. (2012). Molecular Basis for the Activation
of a Catalytic Asparagine Residue in a Self-Cleaving Bacterial Autotransporter. J. Mol. Biol..

[70] Malwal S. R., Gao J., Hu X., Yang Y., Liu W., Huang J. W., Ko T. P., Li L., Chen C. C., O’Dowd B., Zhang Y., Khade R. L., Zhang Y., Oldfield E. (2018). Catalytic Role of Conserved Asparagine, Glutamine,
Serine, and Tyrosine
Residues in Isoprenoid Biosynthesis Enzymes. ACS Catal..

[71] Leferink N. G. H., Ranaghan K. E., Battye J., Johannissen L. O., Hay S., van der Kamp M. W., Mulholland A. J., Scrutton N. S. (2020). Taming the Reactivity of Monoterpene Synthases To Guide
Regioselective Product Hydroxylation. ChemBioChem.

[72] Neumann P., Tittmann K. (2014). Marvels of enzyme catalysis
at true atomic resolution:
distortions, bond elongations, hidden flips, protonation states and
atom identities. Curr. Opin. Struct. Biol..

[73] Cheng L., Bo Z., Krohn-Hansen B., Yang Y. (2025). Directed Evolution and Unusual Protonation
Mechanism of Pyridoxal Radical C–C Coupling Enzymes for the
Enantiodivergent Photobiocatalytic Synthesis of Noncanonical Amino
Acids. J. Am. Chem. Soc..

[74] Claeyssens F., Harvey J. N., Manby F. R., Mata R. A., Mulholland A. J., Ranaghan K. E., Schütz M., Thiel S., Thiel W., Werner H. J. (2006). High-AccuracynComputation
of Reaction Barriers in Enzymes. Angew. Chem.,
Int. Ed..

