# Supplementary material for: Mechanistic Insights into GTP Hydrolysis by the RhoA Protein: Catalytic Impact of Glutamine Tautomerism
Source: ACS Catal. 2025 Feb 28;15(6):4415–28. doi: 10.1021/acscatal.5c00719 (PMC12128172; doi:10.1021/acscatal.5c00719)
Supplement: Supplementary file 1 [file cs5c00719_si_001.pdf]

Supporting Information

Mechanistic Insights into GTP Hydrolysis by the RhoA Protein: Catalytic Impact of Glutamine Tautomerism

Jorge Pardos¶, Adrián García-Martínez, J. Javier Ruiz-Pernía*, Iñaki Tuñón*

Departamento de Química Física, Universidad de Valencia, 46100 Burjassot (Spain)

¶Current address: Instituto de Biocomputacion y Física de Sistemas Complejos (BIFI), Facultad de Ciencias, Universidad de Zaragoza, 50009 Zaragoza (Spain)

*To whom correspondence should be addressed:

Ignacio.tunon@uv.es

j.javier.ruiz@uv.es

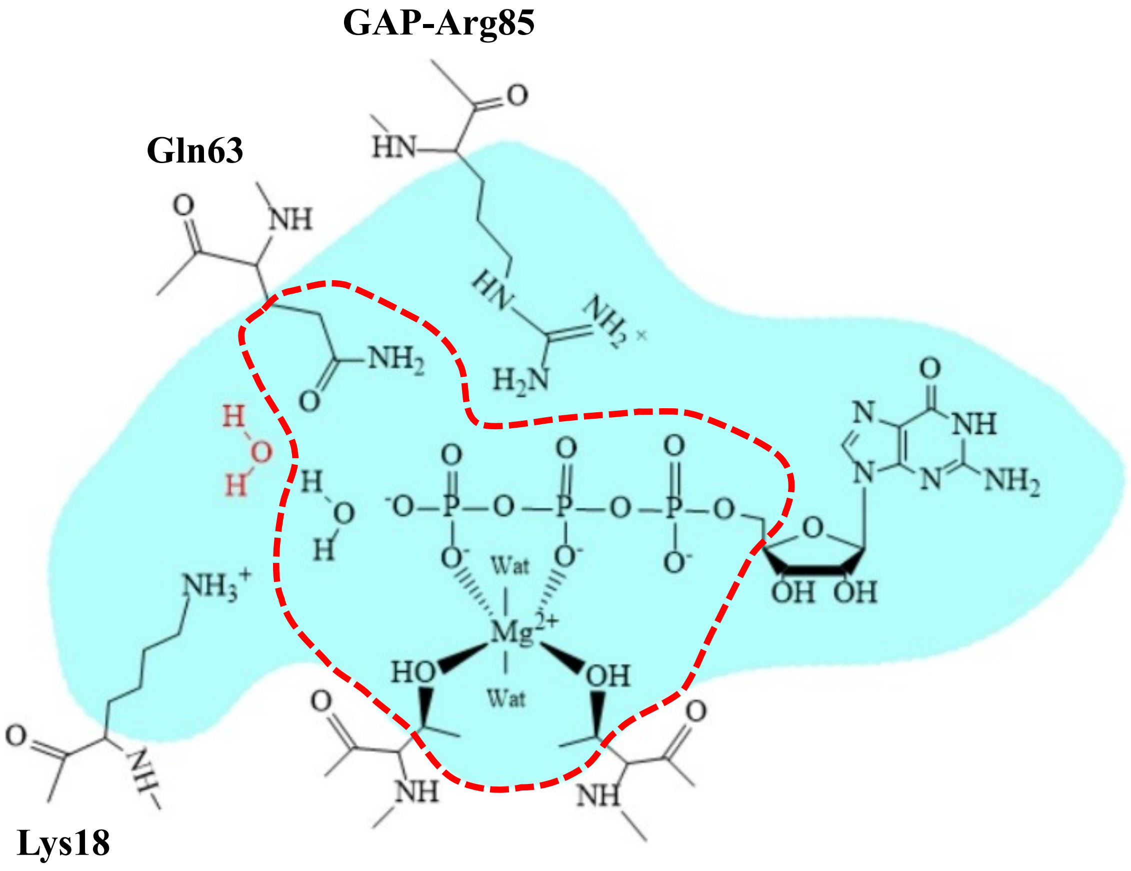

Figure S1. QM region (highlighted in pale blue) used to describe GTP hydrolysis in RhoA-p50RhoGAP at the DFTB3/MM level. The second water molecule (in red) is only included for mechanisms that require it. The QM region used in B3LYPD3/MM simulations is surrounded by a dashed red line.

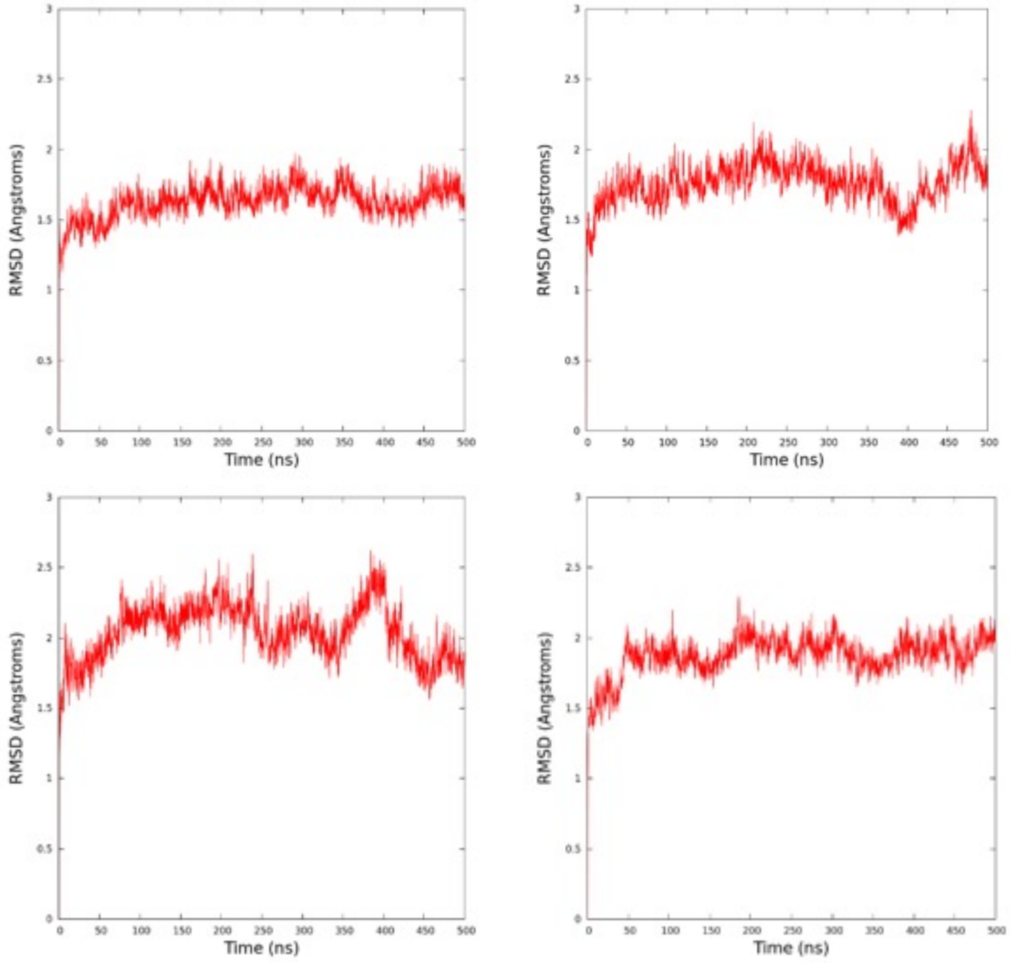

Figure S2. RMSD obtained for the Cα atoms of the RhoA-p50RhoGAP system during the four independent 500 ns MD simulations.

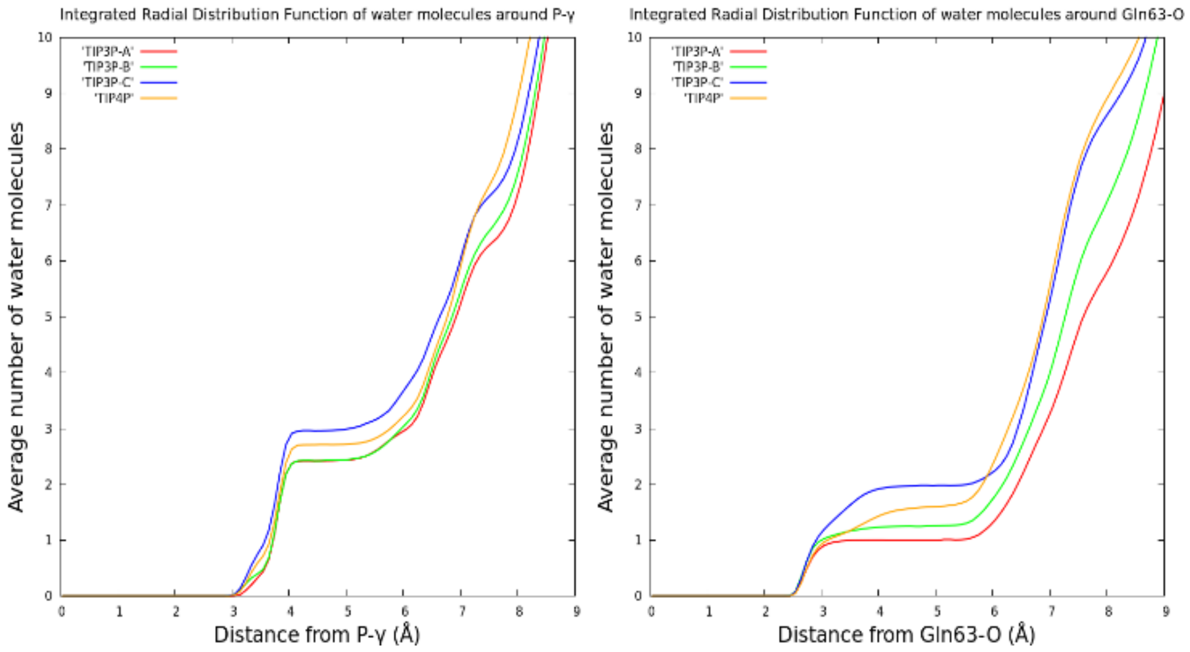

Figure S3. Integrated radial distribution functions of water molecules' oxygen atoms around the Pg atom of GTP (left) and the side chain carbonyl oxygen atom of Gln63 (right). The four representations correspond to three replicas where water was modelled at the TIP3P level (red, green and blue curves) and one replica at the TIP4P level (yellow curve).

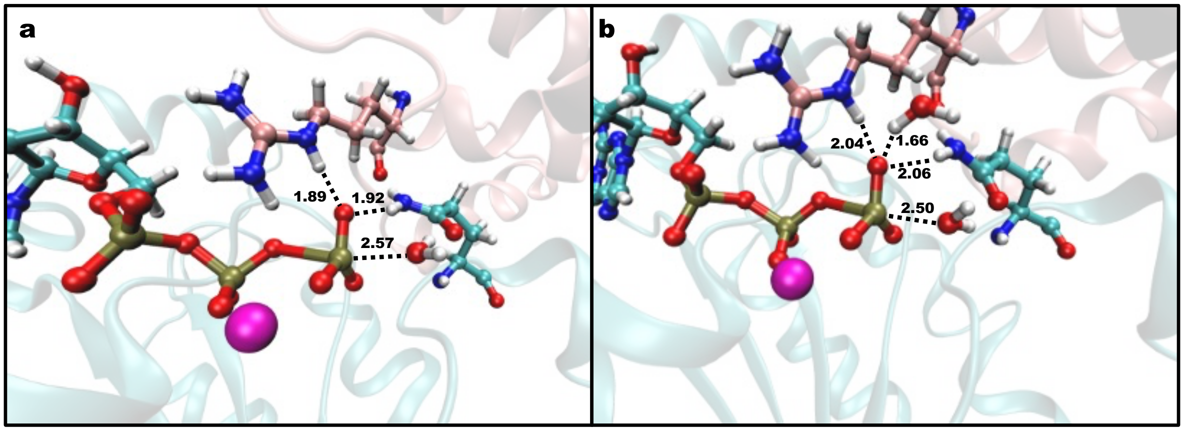

Figure S4. Comparison of the interactions established by the transient metaphosphate anion in the active site during the solvent-assisted (a) or the two water molecules (b) mechanisms.

Data availability

In the following GitHub repository, the PDB files for the structures corresponding to the rate-limiting step of each mechanism are available. The files are named FigureX-TSX.pdb, aligned with the figure references in the paper. Additionally, the structures of reactants and products for the amide-imide mechanism with phosphate-assisted regeneration, computed at the B3LYP level, are provided. The repository can be accessed at: https://github.com/emedio/RhoA-p50RhoGAP
